# Cadmium and Copper Cross-Tolerance. Cu^+^ Alleviates Cd^2 +^ Toxicity, and Both Cations Target Heme and Chlorophyll Biosynthesis Pathway in *Rubrivivax gelatinosus*

**DOI:** 10.3389/fmicb.2020.00893

**Published:** 2020-06-03

**Authors:** Anne Soisig Steunou, Anne Durand, Marie-Line Bourbon, Marion Babot, Reem Tambosi, Sylviane Liotenberg, Soufian Ouchane

**Affiliations:** Université Paris-Saclay, CEA, CNRS, Institute for Integrative Biology of the Cell (I2BC), Gif-sur-Yvette, France

**Keywords:** *CadA*/ZntA, cadmium/copper, metal homeostasis, metal toxicity, cross-talk, [4Fe-4S], porphyrin biosynthesis

## Abstract

Cadmium, although not redox active is highly toxic. Yet, the underlying mechanisms driving toxicity are still to be characterized. In this study, we took advantage of the purple bacterium *Rubrivivax gelatinosus* strain with defective Cd^2 +^-efflux system to identify targets of this metal. Exposure of the Δ*cadA* strain to Cd^2 +^ causes a decrease in the photosystem amount and in the activity of respiratory complexes. As in case of Cu^+^ toxicity, the data indicated that Cd^2 +^ targets the porphyrin biosynthesis pathway at the level of HemN, a *S*-adenosylmethionine and CxxxCxxC coordinated [4Fe-4S] containing enzyme. Cd^2 +^ exposure therefore results in a deficiency in heme and chlorophyll dependent proteins and metabolic pathways. Given the importance of porphyrin biosynthesis, HemN represents a key metal target to account for toxicity. In the environment, microorganisms are exposed to mixture of metals. Nevertheless, the biological effects of such mixtures, and the toxicity mechanisms remain poorly addressed. To highlight a potential cross-talk between Cd^2 +^ and Cu^+^ -efflux systems, we show (i) that Cd^2 +^ induces the expression of the Cd^2 +^-efflux pump *CadA* and the Cu^+^ detoxification system CopA and CopI; and (ii) that Cu^+^ ions improve tolerance towards Cd^2 +^, demonstrating thus that metal mixtures could also represent a selective advantage in the environment.

## Introduction

Metal accumulation, through environmental contamination by anthropogenic release, results in toxicity leading to impaired growth of microorganisms (Nunes et al., 2016). Indeed, excess metal can affect and disrupt different cellular metabolic pathways. Because of their thiophilicity, metals can compete or displace each other in the binding sites of metalloproteins. For instance, copper (Cu^+^), zinc (Zn^2 +^) or cadmium (Cd^2 +^) represent a standing threat for Fe-S proteins in bacteria. They can either directly damage exposed [4Fe-4S] clusters (Macomber and Imlay, 2009) or inhibit components of the iron-sulfur (Fe-S) biogenesis machinery (Tan et al., 2017; Roy et al., 2018; Li et al., 2019). In *Escherichia coli*, Cu^+^, Zn^2 +^or Cd^2 +^ exert their toxic effects by disrupting the solvent exposed [4Fe-4S] clusters of dehydratases (Macomber et al., 2007; Macomber and Imlay, 2009; Xu and Imlay, 2012). These cations can also compete and prevent iron or iron-sulfur cluster binding in IscA, IscU or ferredoxin in the iron-sulfur cluster biogenesis system (Tan et al., 2017; Roy et al., 2018; Li et al., 2019).

In the human pathogen *Neisseria gonorrhoeae*, and the photosynthetic bacterium *Rubrivivax (R.) gelatinosus*, accumulation of Cu^+^ in the cytoplasm affect heme biosynthesis (Azzouzi et al., 2013; Djoko and McEwan, 2013). A hypothesis put forward is that Cu^+^ excess targets HemN, the anaerobic coproporphyrinogen III Oxidase. This enzyme has a conserved cysteine-rich motif, CxxxCxxC, which coordinates a solvent [4Fe–4S] cluster with an iron that anchors a S-adenosylmethionine (SAM) moiety (Layer et al., 2003). It was suggested that Cu^+^ targets this solvent exposed SAM-[4Fe–4S] cluster, thereby affecting the enzyme activity (Azzouzi et al., 2013; Djoko and McEwan, 2013).

Given the importance of Fe-S clusters and heme metabolism in bacteria but also in eukaryotes, the increase of metal concentration in the environment is therefore challenging to all living organisms. Homeostasis maintenance and detoxification of metal excess are therefore crucial to enable bacterial survival in contaminated environments or in macrophages for pathogenic bacteria (Arguello et al., 2007; von Rozycki and Nies, 2009; Capdevila et al., 2017; Chandrangsu et al., 2017).

The intracellular concentration of metals is tightly regulated at the uptake, storage and/or excretion level (Arguello et al., 2007; von Rozycki and Nies, 2009; Capdevila et al., 2017; Chandrangsu et al., 2017). For metals such as copper, cadmium or silver, efflux pumps are effective detoxification systems that allow bacteria to deal with excess metals in their immediate environment (Arguello et al., 2007; von Rozycki and Nies, 2009; Capdevila et al., 2017; Chandrangsu et al., 2017). Indeed, most free-living bacteria possess efflux system to remove metal excess. Members of P_1__*B*_-type ATPases family of heavy metal transporters are universally present in bacteria. They are part of the large superfamily of ATP-driven pumps involved in metal transport across bacterial inner membrane (Arguello et al., 2007). These transporters extrude excess or toxic metal ions such as Cu^+^, Zn^2 +^, Cd^2 +^, or Ag^+^ from the cytoplasm to the periplasm in which metal is handled by other detoxifying proteins (Arguello et al., 2007). Cu^+^, Zn^2 +^, or Cd^2 +^ efflux ATPase mutants accumulate these cations in their cytoplasm (Legatzki et al., 2003; Gonzalez-Guerrero et al., 2010; Djoko and McEwan, 2013; Lu et al., 2016) and very often display growth inhibition phenotype.

A strain of *R. gelatinosus* (TN414) efficient at removing Cd^2 +^ and Zn^2 +^ from cadmium and zinc contaminated soil has been isolated from paddy fields (Sakpirom et al., 2017). *R. gelatinosus* S1 can also grow in medium containing up to 3 mM of cadmium. The mechanisms governing Cd^2 +^ tolerance are however, not yet known. In this study, we first identified the Cd^2 +^-efflux system to ask which pathways Cd^2 +^, a divalent non-redox active cation, impacts in the absence of an efficient efflux system. Interestingly, it is also shown that Cd^2 +^ induces Cu^+^ homeostasis system and that Cu^+^ alleviates Cd^2 +^ toxicity in *R. gelatinosus*, a phenomenon that could represent a selective advantage for bacteria growing in multiple metal-polluted environment.

## Materials and Methods

### Bacterial Strains and Growth

*Escherichia coli* was grown at 37°C in LB medium. *R. gelatinosus* (S1) was grown at 30°C, in the dark aerobically (high oxygenation: 250 ml flasks containing 20 ml medium) and microaerobically (low oxygenation: 50 ml flasks filled with 50 ml medium) or in light (photosynthetically in filled tubes with residual oxygen in the medium) in malate growth medium. Antibiotics were used at the following concentrations: kanamycin (Km), ampicillin and trimethoprim (Tp) at 50 μg/ml, tetracycline at 2 μg/ml. Bacterial strains and plasmids are listed in Supplementary Table SI. Growth inhibition curves were monitored at OD_680__*nm*_ with measurements taken every 15 min for 24 h using a Tecan Infinite M200 luminometer (Tecan, Mannerdorf, Switzerland) for aerobic condition. For photosynthesis conditions, strains were grown as described above and OD was measured after 24 h using the Tecan luminometer. Inhibition growth experiments were done in triplicates starting from a three diluted culture (OD_680_ at 0.02).

### Gene Cloning and Plasmid Constructions for Allele Replacement

Standard methods were performed according to Sambrook et al. (1989) unless indicated otherwise. To inactivate *cadA*, a 1.8 kb fragment was amplified using the primers cadAF1 and cadAR1 (Supplementary Table SII) and cloned into the PCR cloning vector pGEM-T to give pG*cadA*. *cadA* gene was inactivated by deletion of 0.8 kb fragment and the insertion of the Tp cassette at the *Nar*I sites within the *cadA* coding sequence. The resulting recombinant plasmid was designated pG*cadA:Tp*. Furthermore, a plasmid (pB106) containing the full *cadA* and *cadR* genes was also isolated from the DNA library. A 6.6 kb fragment containing *cadA* was subcloned from pB106 in pBBR1MCS-2 at the *Kpn*I-*Sac*I sites. The resulting plasmid was designated pBK*CadA*. *cadR* was PCR cloned using primers cadRF1 and cadRR1 in pGemT plasmid. To inactivate *cadR*, the 1.2 kb Km cassette was inserted in the *Stu*I site within *cadR* in the pG*cadR* plasmid. To generate HemN-H6 fusion, hemN was amplified from wild-type genomic DNA using primers hemN-*Nde*I and hemN-*Xho*I and cloned in the corresponding sites within pET-28b.

### Gene Transfer and Strain Selection

Transformation of *R. gelatinosus* cells was performed by electroporation (Ouchane et al., 1996). The plasmid *pGcadA:Tp* was used to transform the wild-type, *copA*^–^, Δ*copI*, or *copA-H_6_* strains. Transformants were selected on malate plates supplemented with appropriate antibiotics under aerobic conditions. Following transformant selection, template genomic DNA was prepared from the ampicillin sensitive transformants and confirmation of the antibiotic resistance marker’s presence at the desired locus was performed by PCR. Finally, selected colonies were spotted on metal containing plates to confirm their metal sensitive phenotype. Plasmid p*hemN-H_6_* was used to transform the wild-type strain to generate the HemN-H_6_ strain. Clones were selected on kanamycin plates.

### Membrane and Soluble Protein Preparation

The membranes or solubles fractions were prepared by cell disruption with a French press in 0.1 M sodium phosphate buffer (pH 7.4) containing 1 mM phenylmethylsulfonyl fluoride, followed by differential centrifugation. Samples were subjected to a low speed centrifugation step (25, 000 g, 30 min, 4°C) and supernatants were subjected to ultracentrifugation (200, 000 g, 1h30, 4°C) to purify the soluble proteins and collect the membrane fraction in the pellets.

The membranes were then resuspended in the same buffer. Protein concentration was estimated using the bicinchoninic acid assay (Sigma) with bovine serum albumin as a standard. Periplasmic fractions were purified as previously described by Durand et al. (2015).

### Spectrophotometric Measurements

Absorption spectroscopy was performed with a Cary 500 spectrophotometer. For difference spectra (reduced minus oxidized), total pigments were extracted from cell pellets or membranes with acetone-methanol (7/2 [vol/vol]). For each sample, the spectrum was collected on oxidized sample upon addition of 50 μM K_6_Fe(CN)_3_. The sample was reduced by addition of dithionite (few crystals), and the spectrum was collected to generate the reduced minus oxidized spectrum.

### Blue-Native Gel Electrophoresis

To assay *cbb*_3_ and succinate dehydrogenase activities, wild-type and Δ*cadA* strains were grown microaerobically. Membranes were prepared as previously described (Khalfaoui Hassani et al., 2010). Blue-native polyacrylamide gel (BN-PAGE) electrophoresis and in gel-Cox activity assays (DAB staining) were performed as described in (Khalfaoui Hassani et al., 2010) and succinate dehydrogenase activity was assayed using succinate and NBT (Nitroblue tetrazolium) as described for the succinate-NBT reductase assay in reference Wittig et al. (2007).

### Western Blot and Immunodetection

Equal amount of proteins or disrupted cells (OD_680__*nm*_ = 1) were separated by SDS–PAGE (15% polyacrylamide) and further transferred to a Hybond ECL PVDF membrane, (GE Healthcare). Coomassie blue of the SDS gels are shown in Supplementary Material. Membranes were then probed with the HisProbe-HRP (from Pierce) according to the manufacturer instruction and positive bands were detected using a chemiluminescent HRP substrate according to the method of Haan and Behrmann (2007). Image capture was performed with a ChemiDoc camera system (Biorad).

## Results

### The P-type ATPase CadA Is the Primary Cadmium Tolerance Element in *R. gelatinosus*

**FIGURE 1:**
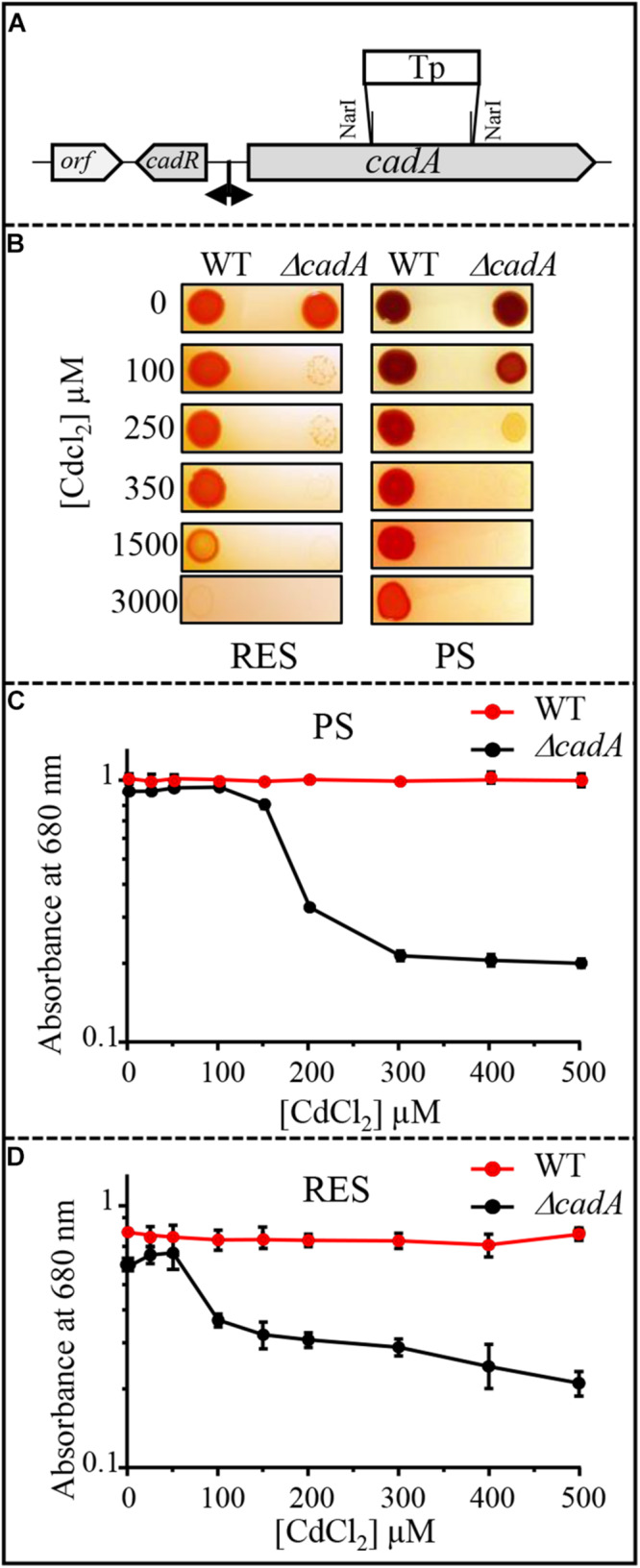
**(A)** Genetic organization of the *cadAR* locus in *R. gelatinosus.* To inactivate *cadA*, the *Nar*I fragment was replaced by antibiotic resistance Tp cassette. **(B)** Growth phenotype of the wild-type (WT) and Δ*cadA* on solid medium supplemented with CdCl_2_. Plates were incubated under aerobic respiration (RES) in the dark or under photosynthesis (PS) for 24h at 30°C prior to photography. **(C,D)** Growth inhibition of the WT and Δ*cadA* mutant challenged with increasing CdCl_2_ concentrations under photosynthesis (PS) **(C)** and respiration (RES) **(D)** conditions. Cells were grown for 24 h at 30°C before OD_680__*nm*_ measurement. The error bars represent the standard deviation of the mean of 3 independent experiments.

Cadmium tolerance in bacteria usually involves the metal efflux pump P_1__*B*_-type ATPase CadA (ZntA) protein that translocates cadmium from the cytoplasm to the periplasm (Rensing et al., 1998). A gene (hereafter referred to as *cadA*) encoding a putative ATPase involved in Zn^2 +^ or Cd^2 +^ efflux was identified in *R. gelatinosus* genome. Sequence analyses showed high similarities with putative heavy metal translocating P_1__*B*_-type ATPases from β-proteobacteria (Supplementary Figure S1). In addition to conserved structural features and motifs in the ATPase sequence, an N-terminal 30 amino acids stretch consisting of 14 histidines and 7 acidic residues, flanked by two CxxxC motifs were found in *R. gelatinosus* CadA (Supplementary Figure S1). This N-terminal cytoplasmic stretch could play a role in metal binding and transport regulation as reported for other metal transporting ATPases (Mana-Capelli et al., 2003; Baekgaard et al., 2010). A gene (referred to as *cadR*) encoding a MerR regulator protein (CadR) was found upstream *cadA*. To ascertain the role of CadA in Cd^2 +^ tolerance, the Δ*cadA* deletion strain was generated and its ability to grow on solid medium in presence of CdCl_2_ was tested in comparison with wild-type strain under aerobic respiration and photosynthesis conditions (Figure 1). When challenged with Cd^2 +^, the growth of Δ*cadA* strain is inhibited with 100 or 250 μM CdCl_2_ under aerobic or photosynthesis conditions, respectively. In contrast, the wild-type strain was still able to grow in presence of 1.5 or 3 mM CdCl_2_ (Figure 1B). The tolerance towards Cd^2 +^ was restored by the expression of the wild-type copy of *cadA* gene *in trans* in Δ*cadA* strain (Supplementary Figure S2). Growth inhibition by Cd^2 +^ of these strains was also assessed in liquid medium in the presence of increasing CdCl_2_ concentration. Whereas growth of the wild-type strain remained unaffected under both conditions, growth of Δ*cadA* mutant was reduced starting at 150 μM or 50 μM CdCl_2_ under photosynthesis (Figure 1C) and respiration (Figure 1D) conditions, respectively. Δ*cadA* mutant was also sensitive to excess Zn^2 +^ as shown by growth inhibition on plates of the wild-type and Δ*cadA* grown in the presence of CdCl_2_, ZnSO_4_ or CuSO_4_ (Supplementary Figure S3A). Similarly, dose response and growth curves in liquid media (Supplementary Figures S3B–D) confirmed that *cadA* was also required for full zinc resistance. We should note that Δ*cadA* was more tolerant to Zn^2 +^ than Cd^2 +^ probably because under Zn^2 +^ excess, Zn^2 +^ uptake (ZnuABC transporter) could be limited by Zur regulator and other Zn^2 +^-efflux systems like CzcCBA could mediate Zn^2 +^ efflux (Arguello et al., 2007; von Rozycki and Nies, 2009; Capdevila et al., 2017; Chandrangsu et al., 2017). Genes encoding these proteins are present in *R. gelatinosus* genome, but their role was not investigated yet. These data confirmed that *cadA* gene encodes for the Cd^2 +^/Zn^2 +^-efflux ATPase CadA, required for Cd^2 +^ (and involved in Zn^2 +^) tolerance in *R. gelatinosus*, and that the Δ*cadA* mutant is more sensitive to cadmium under respiratory conditions.

### Induction of CadA in Response to Excess Cd^2 +^ Involves the MerR-Type Transcriptional Regulator CadR

**FIGURE 2:**
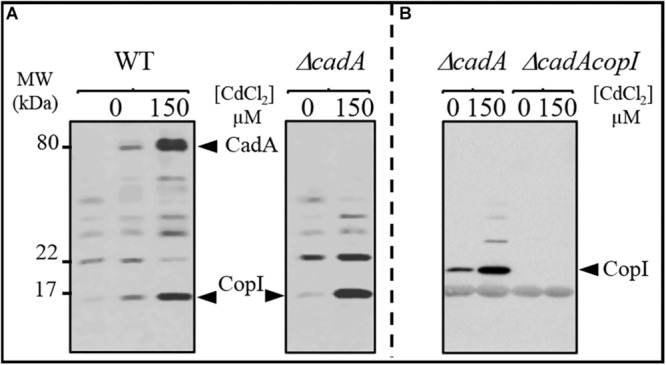
Expression profile of CadA and CopI in *R. gelatinosus* wild-type (WT) and Δ*cadA* cells challenged with Cd^2 +^. Cells were grown overnight (18 h) under photosynthesis and total protein extract from the same amount of cells (OD_680__*nm*_ = 0.1) were separated on a 15% Tris-glycine SDS-PAGE **(A)**. Periplasmic fractions were purified from Δ*cadA* and Δ*cadA_*Δ*copI* cells challenged or not with Cd^2 +^ and separated on a 15% Tris-glycine SDS-PAGE **(B)**. Proteins were visualized after Western blotting using the HRP-HisProbe (Pierce).

To cope with excess toxic metal exposure, bacteria usually induce the expression of metal detoxification proteins including the efflux ATPases. To gain better insight into the expression profile of CadA in response to excess Cd^2 +^, we analyzed the expression of CadA by Western blot in the wild-type and Δ*cadA* strains grown under photosynthesis condition in presence of increasing CdCl_2_ concentration. The presence of the histidine stretch within CadA N-terminus sequence allowed the detection of CadA on Western blot using the horseradish peroxidase (HRP)-conjugated HisProbe. As shown in Figure 2A, addition of 150 μM or 1 mM CdCl_2_ to the medium during wild-type growth resulted in a significant increase in the amount of an 80 kDa protein, likely corresponding to CadA. This band was not detected in the Δ*cadA* cells grown without or with the addition of 150 μM CdCl_2_. To confirm that this band was indeed CadA, the Δ*cadA* complemented strain was also subjected to Cd^2 +^ stress and Western blot showed the expression and induction of the 80 kDa band encoded by *cadA* gene on the replicative plasmid (Supplementary Figure S2B). These findings demonstrated that CadA is induced by cadmium Unexpectedly, in addition to CadA induction, Cd^2 +^ also induced an increase in the amount of the periplasmic Cu^+^ tolerance protein CopI (Durand et al., 2015) in both wild-type and Δ*cadA* cells suggesting a putative role of CopI in Cd^2 +^ tolerance and very likely a cross talk between Cu^+^ and Cd^2^^+^ (see below). To unequivocally show that the Cd^2 +^-induced band correspond to CopI, we generated a mutant (Δ*cadA-ΔcopI*) in which *cadA* and *copI* were deleted, and probed the presence of CopI in the periplasmic fractions. As shown in Figure 2B, while CopI was induced in Δ*cadA* under Cd^2 +^ excess, the band was absent in the Δ*cadA_ΔcopI* mutant showing that the cadmium-induced band correspond to CopI.

**FIGURE 3:**
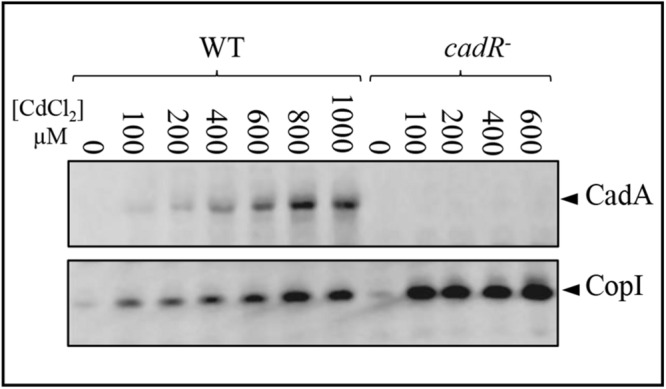
Expression level of CadA and CopI in *R. gelatinosus* wild-type (WT) and *cadR*^-^ mutant cells challenged with increasing concentration of CdCl_2_. Cells were grown overnight (18 h) under photosynthetic condition. Total protein extract from the same amount of cells (OD_680__*nm*_ = 0.1) were separated on a 15% Tris-glycine SDS-PAGE. Proteins were visualized after Western blotting using the HRP-HisProbe.

It has been reported that the transcription regulator CadR/ZntR recognizes a palindromic sequence in the promoter region of *cadA/zntA* genes (Singh et al., 1999). In the intergenic sequence (54 bp) between *cadA* and *cadR* (Figure 1), an inverted repeat sequence (^–^^27^-ACCCGCTACAGGGT-^–^^13^) might allow the binding of regulatory proteins was identified. To further highlight the role of CadR, the gene was inactivated and the resulting *cadR*^–^ strain was shown to be sensitive to cadmium (Supplementary Figure S4) suggesting its involvement in *cadA* expression. To unambiguously show the role of *cadR* in *CadA* expression, we checked the expression level of CadA in the wild-type and the *cadR*^–^ strains challenged with excess Cd^2 +^. For this purpose, cells were grown overnight under photosynthesis in the presence of increasing CdCl_2_ concentration and CadA expression profile was assessed by Western blot. In contrast to the wild-type, overnight growth of *cadR*^–^ cells was inhibited beyond 600 μM CdCl_2_. Western blot analysis confirmed that CadA is induced with increasing concentration of CdCl_2_ (Figure 3) in the wild-type and this induction is CadR-dependent since CadA was not detected in *cadR*^–^ cells. In conclusion, these data showed that the CadA efflux ATPase is required for cadmium detoxification and its expression is induced by CdCl_2_ under the control of CadR. Note that the *cadR*^–^ mutant tolerates a higher cadmium concentration than the Δ*cadA* mutant, but less than the wild-type (Supplementary Figure S4). This difference could be due to a low expression of CadA (undetectable on Western blot) in *cadR*^–^, but sufficient enough to warrant tolerance toward low cadmium concentrations. The Western blot also confirmed the induction of CopI expression in response to Cd^2 +^ stress and showed that induction of CopI by Cd^2 +^ occurred, even in the absence of CadR.

### Cadmium Effect on Photosynthesis and Respiratory Membrane Complexes

**FIGURE 4:**
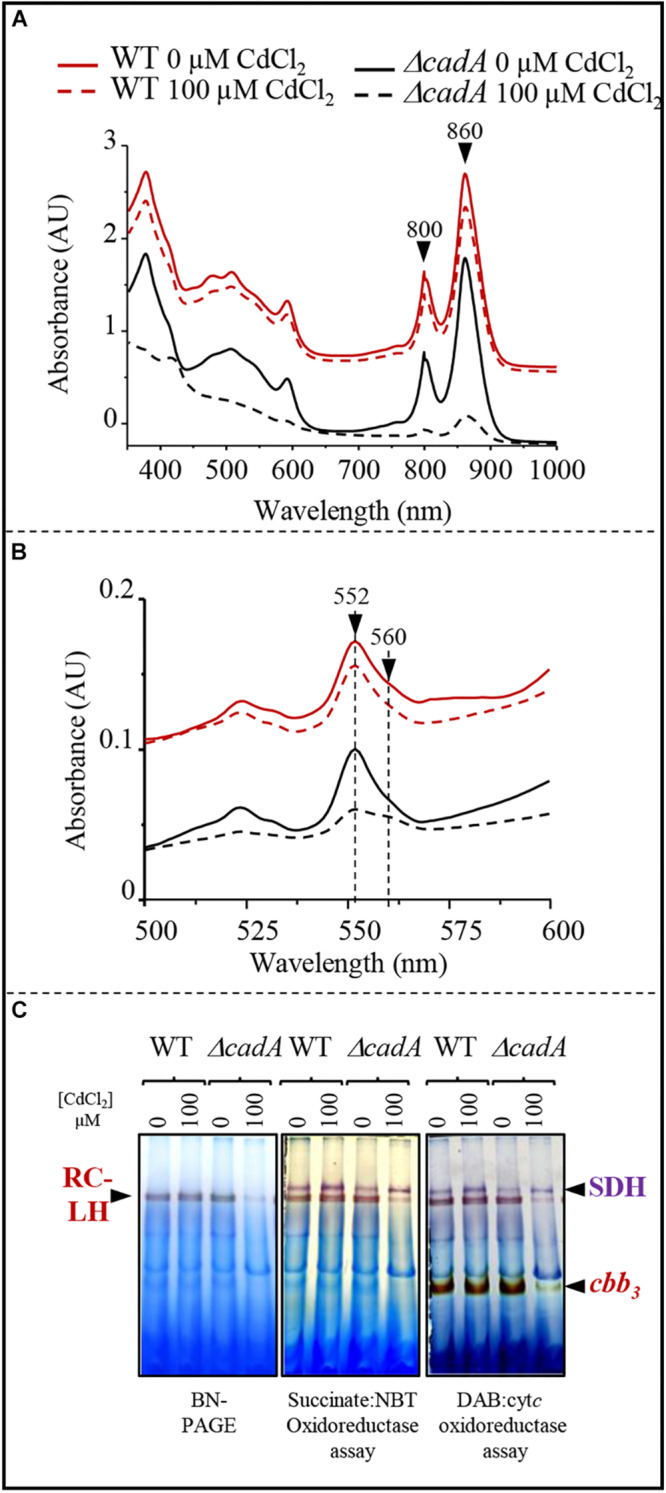
Cadmium effect on photosynthesis and respiratory membrane complexes in the wild-type (WT) and Δ*cadA* mutant grown overnight (18 h) under microaerobic condition. **(A)** Effect of CdCl_2_ on photosystem (860–800 nm) amount in the membranes. **(B)** Difference (reduced minus oxidized) pyridine haemochrome spectra of WT (red) and Δ*cadA* (black) membranes with 100 μM (dashed lines) or without cadmium (lines). **(C)** *cbb*_3_ cytochrome *c* oxidase and succinate dehydrogenase (SDH) *in-gel* activity assays. Equal amount of DDM-solubilized membrane proteins from wild-type (WT) and Δ*cadA* cells grown without or in the presence of 150 μM CdCl_2_ were separated on 3–12% acrylamide-bisacrylamide gradient BN-PAGE. Gels were first assayed for *cbb*_3_ cytochrome *c* oxidase activity (DAB staining) and subsequently assayed for SDH activity (Succinate/NBT). The photosynthetic RC-LH complexes are also visible on the gel thanks to their photopigments.

The toxic effect of Cu^+^ on photosynthesis and respiration in *R. gelatinosus* was previously assessed in the Cu^+^-P_1__*B*_-type ATPase efflux mutant *copA*^–^ (Azzouzi et al., 2013; Liotenberg et al., 2015). Because photosynthetic organisms can also be exposed to Cd^2 +^ in their environment, we took advantage of the Δ*cadA* strain that should likely accumulate Cd^2 +^ in the cytoplasm, to ask whether Cd^2 +^ affects protein complexes and cytochromes involved in photosynthesis and microaerobic respiration. Membranes from the wild-type and Δ*cadA* cells grown microaerobically in medium supplemented or not with 100 μM Cd^2 +^ were enriched and the amount of photosynthetic complexes (RC-LH 860 nm and 800 nm) was assessed with UV-visible absorbance spectra. In contrast to the wild-type, the amount of the photosynthetic complexes was shown to significantly decrease in the membrane of Δ*cadA* cells exposed to Cd^2 +^ (Figure 4A). We also performed redox spectral analyses (reduced minus oxidized spectra) to checked the effect of Cd^2 +^ on cytochromes *c* (peak at 552 nm) and b (peak at 560 nm) in these membranes by comparing the total cytochrome content in the wild-type and Δ*cadA* membranes (Figure 4B). Comparable spectra were recorded for Cd^2 +^ treated and untreated wild-type membrane. Unlike the wild-type, in the Δ*cadA* cells, Cd^2 +^ addition resulted in a drastic decrease of cytochrome *c* and *b* content within the membranes.

BN-PAGE analysis confirmed the decreased amount of reaction centre and light harvesting antenna (RC-LH) in the Cd^2 +^ stressed Δ*cadA* mutant membranes (Figure 4C). Furthermore, cytochrome *c* oxidase *in-gel* assay revealed comparable amount of active cytochrome *c* oxidase *cbb*_3_ in the wild-type membrane from cells grown with or without Cd^2 +^; whereas in Δ*cadA* membrane, the *cbb*_3_ active band decreased significantly in the Cd^2 +^ stressed mutant. Concomitantly, succinate-NBT reductase *in-gel* assay revealed an active band, likely corresponding to SDH, in the wild-type and Δ*cadA* membranes. This band increased slightly in the presence of Cd^2 +^ in both strains. However, the activity was also slightly reduced in Δ*cadA* membranes compared to the wild-type membranes (Figure 4C). The decrease in active cytochrome *c* oxidase *cbb*_3_ was more pronounced than the SDH. The SDH complex requires only one heme per complex while the *cbb*_3_ oxidase requires five hemes for its assembly and activity.

**FIGURE 5:**
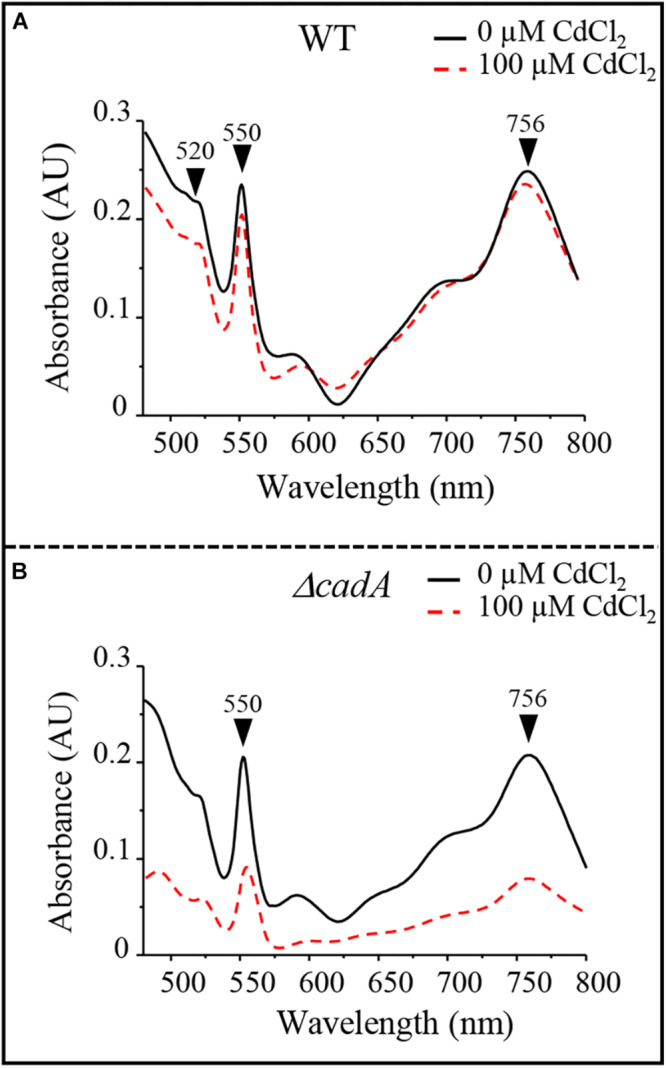
Cadmium effect on total heme (550 nm) and bacteriochlorophyll (756 nm) content in the wild-type (WT) **(A)** and Δ*cadA* strain **(B)** grown by photosynthesis for 18 h in the presence of CdCl_2_.

Photosynthetic and respiratory complexes require chlorophylls and heme cofactors respectively for their assembly and activity. The decreased level of these complexes in presence of excess Cd^2 +^ could be attributed to a decreased amount of cofactors under Cd^2 +^ stress condition in the Δ*cadA* mutant. The total heme and chlorophylls were extracted from cells exposed or not to Cd^2 +^ and dithionite-reduced and ferricyanide-oxidized pyridine hemochrome spectra were recorded. The reduced minus oxidized spectra showed a comparable amount of heme (peaks α and β at 550 nm and 520 nm respectively) and bacteriochlorophylls (756 nm) for photosynthetic grown wild-type strain in presence/absence of Cd^2 +^ in these samples (Figure 5A). The Δ*cadA* mutant cells stressed by 100 μM of Cd^2 +^ presented a strong decrease of the heme and bacteriochlorophylls content compared to the unstressed mutant strain (Figure 5B). Heme and chlorophyll share the same biosynthesis pathway down to protoporphyrin IX. An effect of cadmium on the porphyrin biosynthesis pathway upstream protoporphyrin IX would then affect concomitantly heme and chlorophyll biosynthesis.

### Cadmium Excess Is Associated With an Accumulation of Coproporphyrin III

**FIGURE 6:**
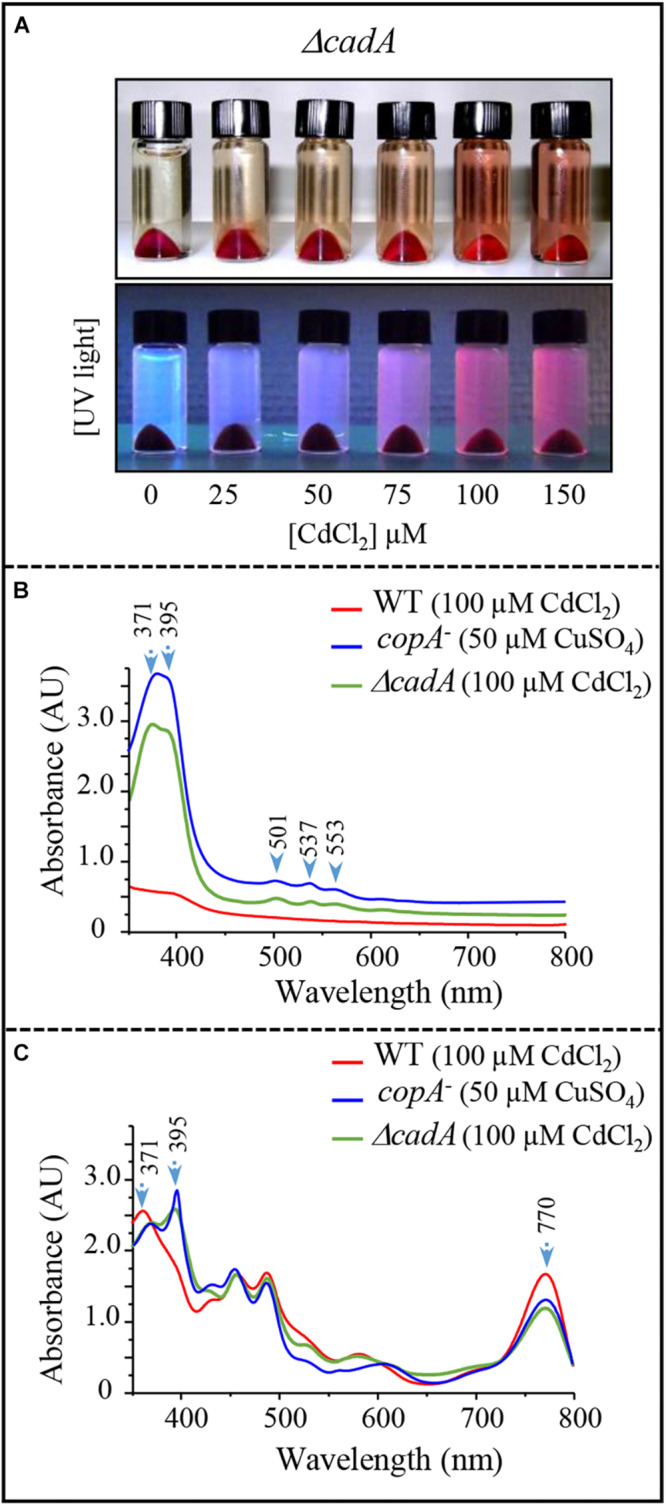
Effect of cadmium on photosynthetic growth in the Δ*cadA* strain and characterization of the pigment extruded in the culture medium. **(A)** Phenotype of the Δ*cadA* mutant grown in the presence of increasing CdCl_2_ concentration. Appearance of coproporphyrin III in the spent medium under visible and UV light. **(B)** UV-visible absorption spectra of the spent medium from the wild-type and Δ*cadA* strain grown with 100 μM CdCl_2_, in comparison with the spent medium of *copA*^–^ strain that accumulates coproporphyrin III (395–371 nm) when grown in the presence of 50 μM CuSO_4_. **(C)** Absorption spectra of total pigment extract from membranes of the wild-type, Δ*cadA* cells grown with 100 μM CdCl_2_ and *copA*^–^ cells grown with 50 μM CuSO_4_. The spectra show the decrease in the amount of bacteriochlorophyll *a* (770 nm) in Δ*cadA* and *copA*^–^ mutants and reveal the presence of coproporphyrin III in these cells.

The wild-type strain and the Δ*cadA* mutant were subjected to increasing sub-lethal concentrations of CdCl_2_ (0–150 μM CdCl_2_) under photosynthetic growth conditions. No effect of excess CdCl_2_ was observed on the wild-type cells (not shown). Conversely, for the Δ*cadA* cells, growth was affected by increasing CdCl_2_ concentration and a UV-fluorescent pigment was released in the spent medium as a CdCl_2_ concentration-dependent manner (Figure 6A). This phenotype also seen under microaerobic conditions, is presumably due to the excretion of porphyrin intermediate and is reminiscent of the *copA*^–^ strain one when exposed to Cu^+^ (Azzouzi et al., 2013). The spent medium of the Δ*cadA* strain was therefore compared to that of wild-type and *copA*^–^ strains using UV-Vis absorbance spectra (Figure 6B). In view of these spectra, the Δ*cadA* strain extrudes the same pigment as the *copA*^–^ strain exposed to Cu^+^. Two absorbance peaks in the Soret region at 371 and 395 nm with minor peaks at 501, 537, and 553 nm were observed. These peaks are characteristic of coproporphyrin III (oxidized coproporphinogen III), a protoporphyrin IX precursor. The presence of coproporphyrin III in the medium may correlate with a decreased amount of chlorophylls and heme in the Δ*cadA* cells exposed to Cd^2 +^. To ascertain this assumption, UV-vis spectra were recorded on total pigments extracted from wild-type and Δ*cadA* strains grown with 100 μM CdCl_2_ or from *copA*^–^ strain grown with 50 μM CuSO_4_ under the same conditions. In both Δ*cadA* and *copA*^–^ strains, the addition of CdCl_2_ or CuSO_4_ respectively to the growth medium correlated with a decrease in the bacteriochlorophyll *a* content (peak at 770 nm) and an increase of the 395 nm peak (Figure 6C). In contrast, in the wild-type strain grown in the presence of 100 μM CdCl_2_, the higher amount of bacteriochlorophyll *a* correlated with the absence of the 395 nm peak in the extract (Figure 6C). These data confirm the accumulation of coproporphyrin III and reflects the decrease of bacteriochlorophyll *a* and heme under photosynthetic and microaerobic conditions in Δ*cadA* cells exposed to Cd^2 +^ as previously reported for the *copA*^–^ mutant exposed to excess CuSO_4_. As for Cu^+^ toxicity, accumulation of coproporphyrin III in the Δ*cadA* strain when stressed with Cd^2 +^ suggested that excess Cd^2 +^ affected the enzyme for which coproporphyrinogen III is a substrate. We therefore assumed that Cd^2 +^ targets the coproporphyrinogen III oxidase HemN in the porphyrin biosynthesis pathway. This is the first *in vivo* demonstration that, as oxygen and Cu^+^, Cd^2 +^ affects the porphyrin biosynthesis pathway presumably at the level of the SAM and [4Fe-4S] containing HemN enzyme.

### HemN Is a Target of Cd^2 +^ and Cu^+^ Toxicity in *R. gelatinosus*

Accumulation of coproporphyrin III in the Δ*cadA* strain when exposed to excess cadmium, or in the CopA defective strains when challenged with excess copper both in *R. gelatinosus* and *N. gonorrhoea*, strongly suggests that HemN activity is affected by excess metal (Azzouzi et al., 2013; Djoko and McEwan, 2013). Inactivating the [4Fe-4S] of HemN by Cd^2 +^ or Cu^+^ would be consistent with the published evidences showing that Cd^2 +^ or Cu^+^ damage exposed [4Fe-4S] in dehydratases (Macomber and Imlay, 2009; Xu and Imlay, 2012). Attempt to express and purify HemN of *R. gelatinosus* and *N. gonorrhoea* (Djoko and McEwan, 2013) in *E. coli* to assess the effect of metal on the enzyme activity were unfortunately unsuccessful. Therefore, to provide direct evidence that Cd^2 +^ or Cu^+^ damage HemN, we hypothesized that the amount of the enzyme would decrease if the [4Fe-4S] cluster is damaged by excess Cu^+^ or Cd^2 +^. To test this assumption, we generated a strain in which the wild-type *hemN* gene was substituted by a histidine tagged copy on the chromosome. In this strain, the *hemN-H_6_* gene is under its own FNR-regulated promoter and should therefore be inducible under micro-aerobic and photosynthesis conditions. This strain was subjected to elevated concentrations of CdCl_2_ or CuSO_4_ and the amount of HemN-H_6_ in the soluble fraction was assessed on Western blot.

**FIGURE 7:**
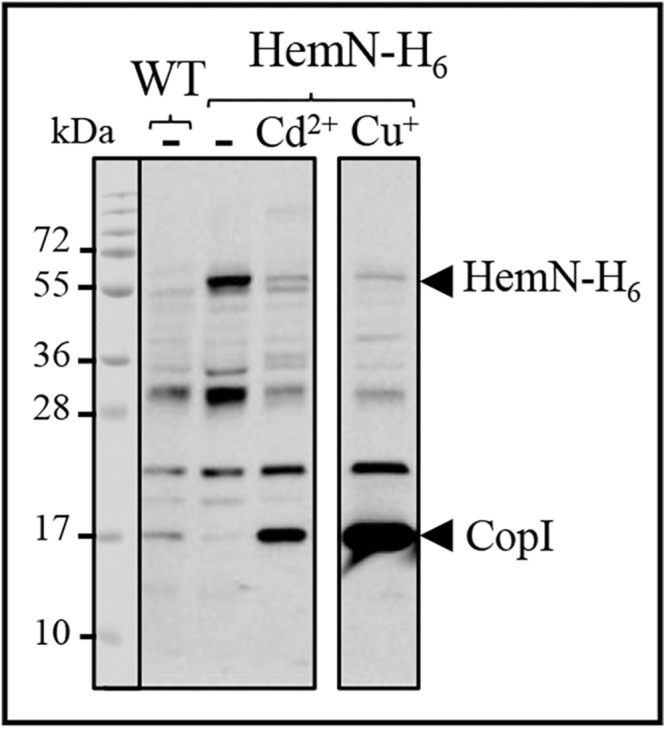
Expression level of HemN-H_6_ in *R. gelatinosus* WT and HemN-H_6_ strain in malate medium (-) or challenged with 1000 μM CdCl_2_ (Cd^2 +^) or 500 μM CuSO_4_ (Cu^+^). Cells were grown overnight (18 h) under photosynthetic condition. Soluble protein fractions (20 μg) were separated on a 15% Tris-glycine SDS-PAGE. Proteins were visualized after Western blotting using the HRP-HisProbe.

A band of 54 kDa likely corresponding to HemN-H_6_ was detected on Western blot in the soluble fraction of HemN-H_6_ strain but not in the wild-type soluble fraction (Figure 7). This band was however significantly decreased in the soluble fractions purified from HemN-H_6_ strain grown in the medium supplemented either with 1000 μM CdCl_2_ or with 500 μM CuSO_4_ (Figure 7). In contrast to the decrease of HemN-H_6_, CopI protein expression was increased in presence of Cd^2 +^ and highly increased by Cu^+^ as expected in these samples (Figure 7), confirming the Cd^2 +^ and Cu^+^ stress status encountered by these cells. These results, clearly demonstrated that *in vivo*, HemN stability is affected by excess Cd^2 +^ and Cu^+^, thus elucidating the effect of metal excess on heme biosynthesis and the cause of coproporphyrin III accumulation.

### Cadmium and Copper Cross-Tolerance: CopI Is Involved in Cadmium Tolerance

On Western Blot, we observed an increase of the periplasmic copper tolerance protein CopI with the addition of CdCl_2_ in both wild-type and Δ*cadA* cells (Figure 2). This CopI induction, in presence of 150 μM CdCl_2_, is higher in the Δ*cadA* mutant than in the wild-type, very likely resulting from Cd^2 +^ accumulation in the cytoplasm of the Δ*cadA* strain. CopI induction by Cd^2 +^ suggested a cross-talk between the Cd^2 +^ and Cu^+^ response systems and a role of CopI and/or the copper detoxification system in Cd^2 +^ response. A band at about 22 kDa apparent molecular weight was also noticeably induced in the Δ*cadA* mutant. This band corresponds to the cytosolic superoxide dismutase SodB (Steunou et al., 2020a). Similarly, in the *cadR*^–^ mutant, CopI is also more induced compared to the wild-type strain. Once again, this difference in expression could arise from the accumulation of Cd^2 +^ in the cytoplasm of *cadR*^–^ cells devoid of the Cd^2 +^-efflux ATPase. The data suggested that either CopR can interact with Cd^2 +^ to induce CopI or that other regulators can induce CopI expression in the *cadR*^–^ strain.

**FIGURE 8:**
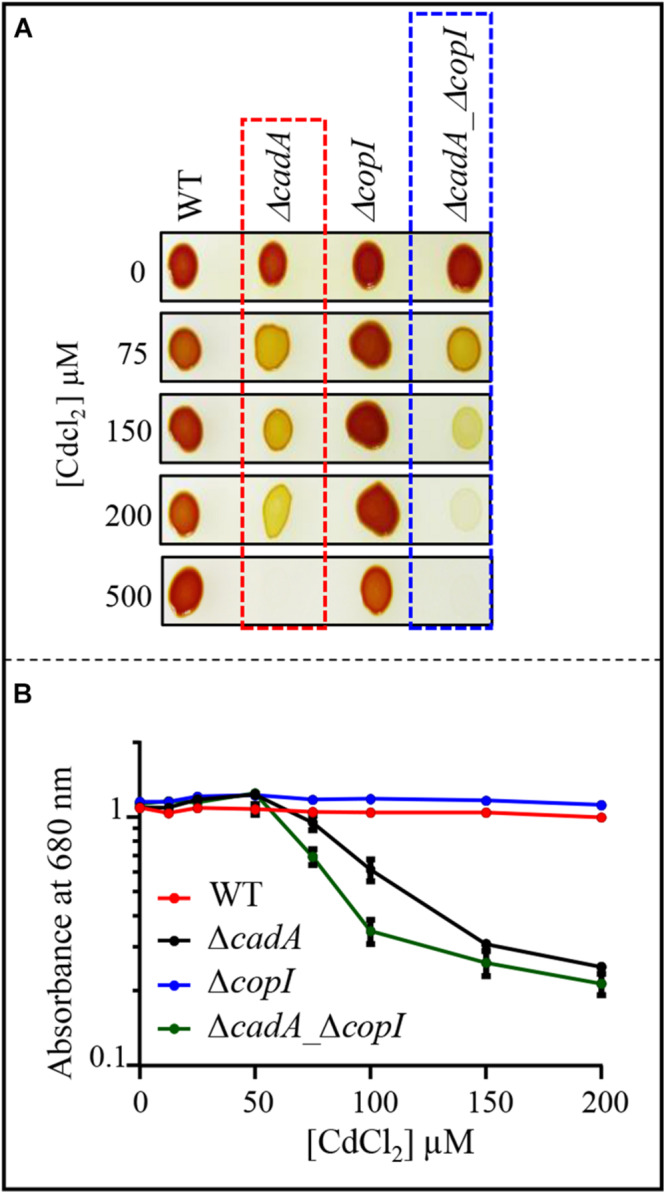
**(A)** Growth phenotype of the wild-type (WT), Δ*cadA*, Δ*copI*, and Δ*cadA*-Δ*copI* mutants in the presence of increasing CdCl_2_ concentrations on solid plates. Cells were grown by photosynthesis for 48 h at 30°C prior to photography. **(B)** Growth inhibition of the wild-type, Δ*cadA, ΔcopI*, and Δ*cadA*_Δ*copI* mutants challenged with increasing CdCl_2_ concentration. Cells were grown in liquid under photosynthetic condition for 21 h at 30°C before OD_680__*nm*_ measurement. The error bars represent the standard deviation of the mean of 3 independent experiments.

Given the induction of CopI, the periplasmic copper tolerance protein, by excess Cd^2 +^ (Figures 2, 3), we wondered whether CopI was required for Cd^2 +^ resistance. The Δ*copI* mutant was sensitive only to high Cd^2 +^ concentration. However, the presence of CadA in the Δ*copI* mutant made the conclusion elusive. To confirm its requirement for Cd^2 +^ tolerance, we question whether inactivation of *copI* in the Δ*cadA* background could give rise to a higher Cd^2 +^ sensitivity in such a strain. The double mutant Δ*cadA_ΔcopI* was constructed and its tolerance towards Cd^2 +^ was compared to that of Δ*cadA* strain on plates (Figure 8A) and liquid (Figure 8B and Supplementary Figure S5) under photosynthesis conditions. The double mutant Δ*cadA_ΔcopI* was indeed more sensitive to Cd^2 +^ than the Δ*cadA* mutant when exposed to 150 μM and 200 μM CdCl_2_ on plates, and to 75 μM CdCl_2_ in liquid, thus confirming that CopI is somehow involved in Cd^2 +^ tolerance. The underlying mechanism is not yet known, however, CopI was shown to be periplasmic and displays conserved His and Met residues that could interact with cations (Durand et al., 2015). It is then tempting to speculate that CopI may handle Cd^2 +^ within the periplasm.

### The Cu^+^-ATPase CopA Is Induced by Cd^2 +^ and Cu^+^ Enhances Cadmium Tolerance in the Δ*cadA* Strain

**FIGURE 9:**
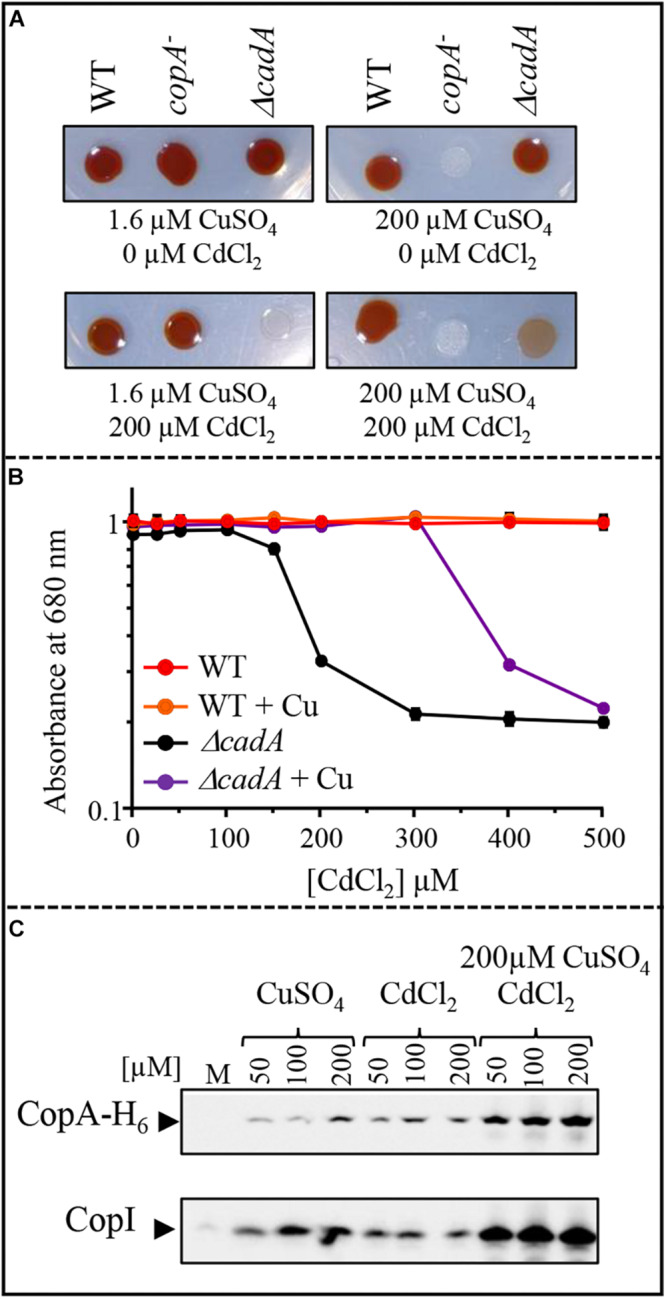
Effect of CuSO_4_ on growth in the presence of increasing concentration of CdCl_2_. **(A)** Growth phenotype of the wild-type (WT), Δ*cadA* and *copA*^–^ strains in presence of increasing CdCl_2_ concentration with or without addition of 200 μM CuSO_4_ on solid plates. **(B)** Growth inhibition of the wild-type and Δ*cadA* mutant challenged with increasing CdCl_2_ concentration in the medium supplemented or not with 200 μM CuSO_4_. Cells were grown under photosynthetic condition for 18 h at 30°C before OD_680__*nm*_ measurement. The error bars represent the standard deviation of the mean of 3 independent experiments. **(C)** Expression level of CopA-H_6_ and CopI in *R. gelatinosus* Δ*cadA-copA-H_6_* mutant challenged with increasing concentration of CuSO_4_, CdCl_2_, or CdCl_2_ + CuSO_4_. Cells were grown in the malate (M) medium supplemented or not with metals, under photosynthetic condition. Total protein extract from the same amount of cells (OD_680__*nm*_ = 0.1) were separated on a 15% tris-glycine SDS-PAGE. Proteins were visualized after Western blotting using the HRP-HisProbe.

Induction of CopI expression in response to both Cu^+^ and Cd^2 +^, suggests a cross-tolerance towards these metals and raised the question on the ability of Cu^+^ to enhance the resistance of Δ*cadA* strain to increasing concentration of Cd^2 +^. To answer this question, we assessed the growth inhibition of Δ*cadA* strain grown under photosynthesis condition in Cd^2 +^ containing medium supplemented or not with 200 μM CuSO_4_. No significant difference in growth of the wild-type strain was observed between the two conditions, both on solid (Figure 9A) and liquid media (Figure 9B). Interestingly, for the Δ*cadA* strain, the addition of CuSO_4_ in the medium significantly improved growth of the mutant, suggesting that, Cu^+^ alleviates Cd^2 +^ stress. Similar results were obtained under aerobic respiration (Supplementary Figure S6).

Given that exposure to Cu^+^ caused an induction of the CopA-CopI system (Azzouzi et al., 2013; Durand et al., 2015), the enhanced growth observed in Δ*cadA* strain could be the result of an up-regulation of CopA and CopI expression by the simultaneous effect of Cu^+^ and Cd^2 +^ in the cell. To check the expression of CopA in response to both Cd^2 +^ and Cu^+^ in the Δ*cadA* background, we constructed a Δ*cadA* strain bearing a *copA* histidine fusion gene (*copA-H_6_*) integrated to the *copA* locus on the chromosome of *R. gelatinosus* to allow detection on Western blot. In this Δ*cadA_copA-H_6_* strain, the transcription of *copA-H_6_* is under the control of its endogenous Cu^+^-regulated promoter. The strain was grown under photosynthesis condition with increasing CuSO_4_ and/or CdCl_2_ concentrations and whole cell extracts were probed for the presence of CopA-H_6_. The presence of both metals resulted in a significant increase in the amount of CopA-H_6_ and CopI within the cells (Figure 8C). These findings clearly showed that as for CopI and CadA, Cd^2 +^ induced the Cu^+^-ATPase CopA. Although transport of Cd^2 +^ by CopA is not yet shown, the data suggest a role of CopA and CopI in Cd^2 +^ tolerance. In agreement with this assumption, we assessed growth on Cd^2 +^ alone or on Cd^2 +^ and Cu^+^ of a Δ*cadA_copA^–^* double mutant devoid of both CadA and CopA ATPases. In contrast to Δ*cadA* single mutant, no difference in growth was observed between the two conditions for the double mutant (Supplementary Figure S7). Together with the involvement of Cd^2 +^ in Cu^+^-homeostasis system induction, these new findings suggested a cross-talk and -tolerance between the Cd^2 +^ and Cu^+^ efflux system in *R. gelatinosus*.

## Discussion

Heavy metal contamination represents a serious threat to the environment and all living organisms because of their acute toxicity. Understanding toxicity is an intricate issue because of the presence of efficient metal detoxification systems; however, the availability of strains defective in a metal homeostasis machinery allowed the identification of new metal targets and shed light on the toxicity mechanisms. Successful efforts have been made to understand the mechanisms by which bacteria respond and deal with single metal excess (Arguello et al., 2007; Barwinska-Sendra and Waldron, 2017; Capdevila et al., 2017; Chandrangsu et al., 2017). In the present study, the Cd^2 +^-efflux ATPase defective mutant of *R. gelatinosus* provides a useful tool to identify targets of Cd^2 +^ within the cells. Exposure of Δ*cadA* to excess Cd^2 +^ resulted in a decreased amount of the light harvesting and reaction center (LH-RC) photosynthetic complexes and a decreased amount of the respiratory complexes succinate dehydrogenase (SDH) and *cbb*_3_ cytochrome *c* oxidase. Both *cbb*_3_ and SDH are hemoproteins, while the LH-RC complex assembly and activity are bacteriochlorophyll dependent. This decrease is concomitant to the decrease in heme and bacteriochlorophyll production. Moreover, noticeably, under microaerobic respiration and photosynthesis growth conditions, the Δ*cadA* mutant extruded coproporphyrin III. These data showed that when challenged with excess Cd^2 +^, the tetrapyrrole biosynthesis pathway was affected at the level of the anaerobic coproporphyrinogen III oxidase HemN. HemN is an [4Fe-4S] cluster-dependent enzyme, with a cluster coordinated only by three cysteines and the methionine group of *S*-adenosylmethionine. This cluster is located within the active site pocket that accommodates two SAM molecules and the coproporphyrinogen III (Layer et al., 2003, 2004). This pocket is likely solvent accessible, making the [4Fe-4S] cluster vulnerable to molecular oxygen but also very likely towards metal ions (Azzouzi et al., 2013; Djoko and McEwan, 2013). The Cd^2 +^-related porphyrin phenotype was observed only under microaerobic respiration or photosynthesis conditions in which HemN is required for the tetrapyrrole biosynthesis pathway, but not under high aeration when the aerobic coproporphyrinogen III oxidase HemF performs the oxidation of coproporphyrinogen III (Breckau et al., 2003). We therefore suggest that Cd^2 +^, like oxygen an Cu^+^, affects tetrapyrrole biosynthesis presumably at the level of the SAM and [4Fe-4S] containing HemN enzyme.

Under aerobic respiration condition, Δ*cadA* growth is even more affected by Cd^2 +^ than under photosynthesis condition, this might be related to the synergetic toxic effects of Cd^2 +^ and oxygen. Yet, the Cd^2 +^ targets remain to be identified in *R. gelatinosus*. Given that Cd^2 +^ targets [4Fe-4S] clusters under microaerobic respiration and photosynthesis conditions, it is tempting to suggest that other oxygen sensitive [4Fe-4S] dehydratases may be poisoned by the simultaneous presence of oxygen and Cd^2 +^, as shown in *E. coli* (Xu and Imlay, 2012) and in *R. gelatinosus* (Liotenberg et al., 2015).

In their environment, bacteria are exposed to a mixture of metals (Ye et al., 2017). This can cause interactions between metals, enhanced toxicity or on the contrary, generate cross-resistance towards different metals. Interestingly, we identified CopI, known to be involved in Cu^+^ tolerance (Durand et al., 2015), as also involved in Cd^2 +^ tolerance since deletion of *copI* in Δ*cadA* mutant resulted in an increased sensitivity to Cd^2 +^. The mechanism by which CopI interferes with Cd^2 +^ tolerance is unknown, however, since CopI interacts with Cu^+^, Cd^2 +^ may also interact with the apo-CopI protein in the absence of Cu^+^. In *P. aeruginosa*, the *pcoAB* genes are induced by Cu^+^ and encode periplasmic Cu^+^ binding proteins. The *pcoAB* genes are also suggested to be involved in Zn^2 +^ and probably Cd^2 +^ tolerance since the *pcoAB* mutants are more sensitive to Zn^2 +^ (Teitzel et al., 2006).

Interaction of Cd^2 +^ with cupro-protein, albeit with low affinity, have been reported in the case of the Cu^+^ chaperone CopZ involved in copper efflux in *Bacillus (B.) subtilis* and other bacteria (Kihlken et al., 2008). As for *copI*, the *copZ* deletion in *B. subtilis* resulted in an increased sensitivity to both Cu^+^ and Cd^2 +^ and expression of the *copZA* operon was induced in response to elevated concentrations of both metals (Moore et al., 2005; Steunou et al., 2020a). Similarly, in *Pseudomonas (P.) aeruginosa* Cu^+^ induces the activation of the *czcCBA* operon involved in the efflux of Zn^2 +^, Cd^2 +^, and Co^2 +^ (Caille et al., 2007). In our study, Cd^2 +^ is shown to induce the Cd^2 +^-efflux ATPase CadA and the Cu^+^-efflux ATPase CopA in *R. gelatinosus*. In addition, Cu^+^ improves Cd^2 +^ tolerance in the CadA deficient mutant, suggesting that CopA and CopI are somehow involved in Cd^2 +^ detoxification. The involvement of CopA in Cd^2 +^ or Zn^2 +^ efflux has never been demonstrated and seems very unlikely. Nevertheless, the cross-talk between Cu^+^ and or Zn^2 +^/Cd^2 +^ efflux systems, at least at the expression level, was previously reported in other bacteria. In *P. aeruginosa*, both PA3920 gene encoding CopA and PA3690 encoding ZntA/CadA homologue are induced by Cu^+^ stress (Teitzel et al., 2006). We should stress out that in this study, *P. aeruginosa* wild type cells were exposed to a very high concentration of CuSO_4_ (10 mM). In a more recent study, Arguello’s group used RNA-Seq to characterize the response of *P. aeruginosa* to 0.5 mM CuSO_4_ to avoid pleiotropic effects that could be related to elevated concentration of CuSO_4_ (Quintana et al., 2017). PA3690 (ZntA/CadA) was not induced in the wild type, nevertheless, in the Δ*cueR* and Δ*copR* mutant, a 3.6 and 2 fold change in the expression of PA3690 was observed in these mutants (Quintana et al., 2017). Likewise, in the filamentous cyanobacterium *Oscillatoria brevis*, expression of the ATPase Bxa1 is also induced by both Cd^2 +^ and Cu^+^ cations (Liu et al., 2004). Cross-tolerance towards Cu^+^ and Zn^2 +^ or Cd^2 +^ involving these ATPases, was not investigated in these bacteria.

Although the mechanism and physiological significance behind Cu^+^/Cd^2 +^ growth improvement reported in this work remain unclear and require further investigation, the cross-talk and cross-tolerance reported in all these studies emphasize on the importance of interactions and toxicity that could arise from metal mixtures and show that research should further consider mixtures effects.

Metal ability to undergo redox reactions is important for its function as protein cofactor but also for its intrinsic toxicity. Cd^2 +^ is not redox active and cannot directly trigger ROS formation, yet, it is very toxic. It was suggested that Cd^2 +^ could indirectly promote ROS production by displacing and releasing redox active ions such as iron or copper from proteins (Xu and Imlay, 2012; Barwinska-Sendra and Waldron, 2017). Induction of SodB expression in the Δ*cadA* strain suggests an increase in ROS production or a dysregulation of iron homeostasis since SodB is known to be regulated by Fur regulator in bacteria. Together with the degradation of solvent exposed [4Fe-4S] cluster from key metabolic enzymes, iron homeostasis dysregulation and ROS should be considered to account for the Cd^2 +^ toxicity in the Δ*cadA* strain (Steunou et al., 2020b).

## Data Availability Statement

The raw data supporting the conclusions of this article will be made available by the authors, without undue reservation, to any qualified researcher.

## Author Contributions

AS, AD, MB, SL, and SO designed the research. AS, AD, M-LB, RT, and SO performed the research. AS, AD, MB, SL, M-LB, and SO analyzed the data. AS and SO wrote the manuscript.

## Conflict of Interest

The authors declare that the research was conducted in the absence of any commercial or financial relationships that could be construed as a potential conflict of interest.

## Abbreviations

BN-PAGE: blue native polyacrylamide gel electrophoresis
DAB: 3,3’-diaminobenzidine tetrahydrochloride
DDM: n-dodecyl - β -D-maltopyranoside
HRP: horseradish peroxidase
LH-RC: light harvesting-reaction center
PVDF: polyvinylidene difluoride.
