# Supplementary material for: Cadmium and Copper Cross-Tolerance. Cu+ Alleviates Cd2 + Toxicity, and Both Cations Target Heme and Chlorophyll Biosynthesis Pathway in Rubrivivax gelatinosus
Source: Front Microbiol. 2020 Jun 3;11:893. doi: 10.3389/fmicb.2020.00893 (PMC7283390; doi:10.3389/fmicb.2020.00893)
Supplement: Supplementary file 1 [file Data_Sheet_1.PDF]

Table SI: Bacterial strains and plasmids

	Relevant characteristics	Source
Strains		
E. coli		
JM109	*el4⁻(McrA⁻), recA1, endA1 gyrA69, thi-1, hsdR17 (rk-mk+) supE44, reA1, Δ(lac-proAB) [F'traD36, proAB, lacIZΔM15*	Stratagene
R. gelatinosus		
Strain S1	Wild-type	(Uffen, 1976)
ΔcadA	*cadA* deleted strain (*ΔcadA::Tp*)	This work
$cadR^-$	*cadR* inactivated strain (*cadR::K*)	This work
$copA^-$	*copA* inactivated strain (*copA::Tn5,Km*)	(Azzouzi et al., 2013)
ΔcopI	*copI* deleted strain (*copI::Km*)	(Durand et al., 2015)
ΔcadA_ΔcopI	*cadA* (*ΔcadA::Tp*) and *copI* (*copI::Km*) deleted strain	This work
*ΔcadA_*$copA^-$	*cadA* (*ΔcadA::Tp*) and *copA* (*copA::Km*) deleted strain	This work
copA-H_6	strain bearing the 6-his tagged *copA* gene on the chromosome	(Azzouzi et al., 2013)
ΔcadA_copA-H_6	*cadA* deleted strain (*ΔcadA::Tp*) bearing the 6-his tagged *copA* gene on the chromosome	This work
hemN-H_6	strain bearing the 6-his tagged *hemN* gene on the chromosome	This work
Plasmids		
pGEM-T	Cloning vector (Ap^r)	Promega
pUC4K	Plasmid bearing the Km cartridge (Ap^r Km^r)	Pharmacia
p34S-Tp	Plasmid bearing the Tp cartridge (Ap^r Tp^r)	(Dennis and Zylstra, 1998)
pBBR1MCS-3	(mob^+, Tc^r) expression vector	(Kovach et al., 1994)
pB106	pBBR1MSC-4 with 6.6 kb insert containing the *cadRA* genes isolated by PCR from WT genomic DNA library	This work
pG*cadA*	pGEMT + 1.8 kb PCR fragment containing *cadA*	This work
pG*cadA::Tp*	Tp cartridge cloned into NarI sites deleting 0.8 kb of *cadA* in *pGcadA*	This work
pG*cadR*	pGemT + 1Kb PCR fragment containing *cadR*	This work
pG*cadRK*	Km cartridge cloned into stuI site inactivating *cadR* in *pGcadR*	This work
pBK*CadA*	6.6 kb KpnI-SacI fragment from pB106 in pBBR1MCS-2	This work
p*copA-*H_6	*copA* gene cloned in pET-28b plasmid at the EcoRI-XhoI site	(Azzouzi et al., 2013)
p*hemN-*H_6	*hemN* gene cloned in pET-28b plasmid at the NdeI-XhoI site	This work

Ap^r, ampicillin resistant, Km^r, kanamycin resistant, Tp^r, trimethoprim resistant, Tc^r, tetracyclin resistant.

Table SII: Primers used in this work

**Primer**	5' to 3' Sequence
cadAF1	GAACGCACGCTGCGCCTGGA
cadAR1	CATCAAGGCGGTGTTCCT
cadRF1	ACGACGACGAGGAGTTCGTCGA
cadRR1	GATCTGCCCTTCCTCAGACGGCGCAG
hemN-NdeI	GGATGAATCATATGGACACGATT
hemN-XhoI	CCCGTGCCGGCTCGAGATGATGCG

References

Azzouzi, A., Steunou, A.S., Durand, A., Khalfaoui-Hassani, B., Bourbon, M.L., Astier, C. et al. (2013) Coproporphyrin III excretion identifies the anaerobic coproporphyrinogen III oxidase HemN as a copper target in the Cu^+-ATPase mutant *copA*$^-$ of *Rubrivivax gelatinosus*. *Mol Microbiol* **88**: 339-351.

Dennis, J.J., and Zylstra, G.J. (1998) Plasposons: modular self-cloning minitransposon derivatives for rapid genetic analysis of gram-negative bacterial genomes. *Appl Environ Microbiol* **64**: 2710-2715.

Durand, A., Azzouzi, A., Bourbon, M.L., Steunou, A.S., Liotenberg, S., Maeshima, A. et al. (2015) c-type cytochrome assembly is a key target of copper toxicity within the bacterial periplasm. *MBio* **6**: e01007-01015.

Kovach, M.E., Phillips, R.W., Elzer, P.H., Roop II, R.M., and Peterson, K.M. (1994) pBBR1MCS: a broad-host-range cloning vector. *BioTechniques* **16**: 800-802.

Uffen, R.L. (1976) Anaerobic growth of a *Rhodopseudomonas* species in the dark with carbon monoxide as sole carbon and energy substrate. *Proc Natl Acad Sci USA* **73**: 3298-3302.
