# Supplementary material for: Cadmium and Copper Cross-Tolerance. Cu+ Alleviates Cd2 + Toxicity, and Both Cations Target Heme and Chlorophyll Biosynthesis Pathway in Rubrivivax gelatinosus
Source: Front Microbiol. 2020 Jun 3;11:893. doi: 10.3389/fmicb.2020.00893 (PMC7283390; doi:10.3389/fmicb.2020.00893)
Supplement: Supplementary file 2 [file Data_Sheet_2.PDF]

Supplementary Material

Cadmium and Copper Cross-tolerance. Cu^{+} alleviates Cd^{2+} toxicity, and both cations target heme and chlorophyll biosynthesis pathway in *Rubrivivax gelatinosus*.

Anne Soisig Steunou, Anne Durand, Marie-line Bourbon, Marion Babot, Reem Tambosi, Sylviane Liotenberg, and Soufian Ouchane*

Université Paris-Saclay, CEA, CNRS, Institute for Integrative Biology of the Cell (I2BC), 91198, Gif-sur-Yvette, France.

* Address correspondence to: soufian.ouchane@i2bc.paris-saclay.fr. Phone: (33)-169823137. Fax: (33)-169823230.

Keywords: CadA/ ZntA, Cadmium/ Copper, Metal Homeostasis, Metal toxicity, Cross-talk, [4Fe-4S], porphyrin biosynthesis.

Running Title: ***Cross-tolerance and targets of metals in bacteria***

```
R.gel      MDTNKTDSDGCGEGCGCTPVAAVALPMPTRRRRVPEPAHEHSHDHGHEHGHDHGHAHTHG
Aci.sp     --------------------------------------------MAHDHPHDIKPDPAGA
Bur.sp     --------------------------------------------MAHDHPQDIQPNRTGT
Bor.sp     ----------------------------------------------MNMPRTQAPTTS--
E.coli     --------------------------------------MSTPDNHGKKAPQFAA-FKPLT
                                                          .  :

R.gel      DGHAHEHDCCGGACAAPTDTTPDPGGPGLKLRIPAMDCAVEEGQIRRALEGIDGIRTLRF
Aci.sp     LQPAGCGACCGASDHAPAASASVPLGGGQRFRIPAMDCASEESEIRRAVEGIPGVLGLRF
Bur.sp     APVPACGPCCGAVEHAPVASVPAPPGGGQRFRIPAMDCASEESEIRRAVEGVPGVLGLRF
Bor.sp     -PSEPASACSGHACTTPTPDAAAATPGWARFRVPTMDCASEESEIRRAVEHIDGIRAMAF
E.coli     TVQNANDCCCDGACSSTPTLSENVSGTRYSWKVSGMDCAACARKVENAVRQLAGVNQVQV
                   *..    :               ::  ****    ::..*:. : *:  : .

R.gel      ALAERTLRLDAEPAARDAALAAIRRAGFAAEPLADDAAP------------LPWWRAAGL
Aci.sp     QLGERSLRIAGEPQAALAAVEAIRNSGFEVHAWPDATESSNAGDGSDHGHD--AATGSGL
Bur.sp     QLGERSLRIAGEPQAALAAVEAIRNTGFEVHAWPDAPESTSAGVGSDHEHEHAATTGSGL
Bor.sp     QLGQRTVAIDAPGTSIEQAIAAIRKAGFDPQPLPTAKAVSTASAGATADDHDLGERASGA
E.coli     LFATEKLVVDADNDIRAQVESALQKAGYSLRDEQAAEEPQASR------------LKENL
            :. ..: : .       .  *::.:*:  .                           .

R.gel      PRLGAALALAAAAELLHALLPASQTAEWAGMALAAAAIALAGLGVL-KKGFAAL-ARRRL
Aci.sp     PRLLAALALATAAEALSFLAPDAPWSKWAGLAVAAAAIGLAGFGTF-RKGLSAL-RHGRL
Bur.sp     PRLLAALALATAAEGLSFLVPDAPWSKWVGLAVAAAAIALAGFSTF-SKGLTAL-RHGRL
Bor.sp     WRLGAALLLAVGAEALGFFAPESQAWKSAGFGLAAAAIWLAGFEVY-KKGLMAL-RHGRL
E.coli     PLITLIVMMAISWGLEQFNHPFGQL--------AFIATTLVGLYPIARQALRLIKSGSYF
             :   : :* .        * .          *  *  *.*:     :.:  :     :

R.gel      DINALMSVAVVGAFAIGQWPEAAMVMALYTIAELIEARAVDRARGAIAALMAMAPEVATL
Aci.sp     NINALMSVAVAGAFVIGQWPEAAMVMALYAIAELIEARAVDRARNAISGLLALSPDTADL
Bur.sp     NINALMSVAVAGAFVIGQWPEAAMVMALYAIAELIEARSVDRARNAISGLLALSPDTADL
Bor.sp     NINALMTVAVTGAFAIGQWPEAAMVMALYAIAEAIEARAVDRARNAIKGLLAMAPEEASV
E.coli     AIETLMSVAAIGALFIGATAEAAMVLLLFLIGERLEGWAASRARQGVSALMALKPETATR
            *::**:**. **: **   *****: *: *.* :*. :..*** .: .*:*: *: *

R.gel      RR-AGAWVTVPVAEAAVGETLRVRPGERVPVDAVLSEGTTSVDQAAVTGESLPVDKQPGD
Aci.sp     RQPDGTWRSAPVGSVPIGATVRVKPGERVAMDAIVTDGSTAVDQAPVTGESLPVDKGPGD
Bur.sp     RQPDGTWKTMPVGDVPIGATVRVRPGERVAMDAVVTEGSTSLDQAPVTGESLPADKGPGD
Bor.sp     RQSDGSWVSRPVAAIPVGALLRVKPGERVPMDGIVRSGQTSINQAPVTGESIPVDKTAGD
E.coli     LR-KGEREEVAINSLRPGDVIEVAAGGRLPADGKLLSPFASFDESALTGESIPVERATGD
            :  *      :     *  :.*  * *:  *. :  .  ::.::: :****:*.::  **

R.gel      ALFAGTLNLSGAVEARITAPASRSTLARIVEAVEHAQASRAPTQRFVDRFAAVYTPAVFA
Aci.sp     AVFAGTINQTGTIECRVTAAASQSTLARIIHAVEQAQGSRAPTQRFVDRFAAVYTPAVFV
Bur.sp     AVFAGTINQTGTIECRVTAAASQSTLARIIHAVEQAQGSRAPTQRFVDRFAAVYTPAVFV
Bor.sp     PVFAATINESGTFEFEVTAPASDSTLSRIIHAVEQAQATRAPTQGFVDRFAAVYTPTIFV
E.coli     KVPAGATSVDRLVTLEVLSEPGASAIDRILKLIEEAEERRAPIERFIDRFSRIYTPAIMA
            : *.: .    .  .: :  . *:: **:. :*.*:  *** : *:***: :***:::.

R.gel      VALAVALGGPLVAGWAWTAAIYKALVLLVIACPCALVISTPVALSSGLAAAARRGVLLKG
Aci.sp     LALAVAIAGPWLFGWTVLAAVYKALVLLVIACPCALVISTPVTVVSGLASAARRGILIKG
Bur.sp     LALAVAIAGPWLFGWTVLAAVYKALVLLVIACPCALVISTPVTVVSGLASAAKRGILIKG
Bor.sp     IALAVAVLGPWLLGWTWMQAVYKALVLLVIACPCALVISTPVTIVSGLAAAARRGILVKG
E.coli     VALLVTLVPPLLFAASWQEWIYKGLTLLLIGCPCALVISTPAAITSGLAAAARRGALIKG
           :** *::  * : . :    :**.*.**:*.**********.:: ****:**:** *:**

R.gel      GAHLEAARRIRAVAFDKTGTLTEGRPPLVHWQTLAGD--EAAARALGAALAAQTDHPVSR
Aci.sp     GVHLEQARLLKAVALDKTGTITEGRPRLVEWEMLSTSLPKPQAEHIAAALAAHSDHPVSR
Bur.sp     GVHLEQARLIKAVALDKTGTITEGKPRLVEWEMLTTSLPRSQAEHIAAALAAHSDHPVSR
Bor.sp     GIYLEEARKLKAIALDKTGTITEGKPRLVDWSVVDPATEPAAAEHIAVVLASHSDHPVSK
E.coli     GAALEQLGRVTQVAFDKTGTLTVGKPRVTAIHPATGIS-ESELLTLAAAVEQGATHPLAQ
           *  **    :  :*:*****:* *:* :.                :...:   : **:::

R.gel      AIAAGLA--PAAPAAVSAVQALHGRGVVAQADGAAVWLGSRRLAAERGALTPELEAALAE
Aci.sp     AIAAGLT--PNSVEA-RNFAALSGRGIEADIGGQRYVLGNHRLIEERGQCSAEIEAVLHR
Bur.sp     AIAAGLT--PNSVEA-RNFSALSGRGIEADIGGQRYVLGNHRLIEERGQCSAEIEAVLHR
Bor.sp     AIAAGLK--PNGVEA-EAFTALAGRGVQGKVGDVTYVLGNHRLIEERGQCSQALEERLKQ
E.coli     AIVREAQVAELAIPTAESQRALVGSGIEAQVNGERVLICAAG-----KHPADAFTGLINE
           **.        .  :     ** * *: .. ..    :            :  :   : .

R.gel      HERQGRGVTLLGRGAQALALFAVADTLKPGAREAVSALKALGVVPVMLSGDHGAAAQAVA
Aci.sp     HEEAGRTVSLLATASGVLALFAVADTIKPSSAAAIGELKAMGITPVMLTGDNQATAEAIG
Bur.sp     HEAAGRTVSLLATASGVLALFAVADTIKPSSAAAIGELKAMGITPVMLTGDNQATAEAIG
Bor.sp     HEDAGRTVTLLATPEKVIALFAVADTIKPSSREAVAELRALGITPVMLTGDNQATASAVA
E.coli     LESAGQTVVLVVRNDDVLGVIALQDTLRADAATAISELNALGVKGVILTGDNPRAAAAIA
            *  *: * *:     .:.::*: **:: .:  *:. *.*:*:  *:*:**:  :* *:.

R.gel      AEAGIAEVRADLLPEDKLAAIEALQRRHGLTAMAGDGLNDAPALARSDLGIAMGAAGTDV
Aci.sp     REAGMTDIRGNLLPEDKLAAIQAMQKQYGATAMTGDGINDAPALAQADIGVAMGAAGTDI
Bur.sp     REAGLTDIRGNLLPEDKLAAIQAMQKQYGATAMTGDGINDAPALAQADIGVAMGAAGTDI
Bor.sp     HEAGIEQARGNLLPEHKLEAIKALQREIGLTAMTGDGINDAPALAQADIGVAMGAAGTDT
E.coli     GELGLE-FKAGLLPEDKVKAVTELNQ-HAPLAMVGDGINDAPAMKAAAIGIAMGS-GTDV
            * *:   :..****.*: *:  :::  .  **.***:*****:  : :*:***: ***

R.gel      AIEAADVVVMNDDPRRVAETVRLSRATHAVLVQNIVLALGIKAVFLALAVFDDATMWMAV
Aci.sp     AMEAADVVVMNDDLRRIAETVRLSQRTHAVLWQNIALALGIKAVFLVLALFDNASMWMAV
Bur.sp     AMEAADVVVMNDDLRRIAETVRLSQRTHAVLWQNIVLALGIKAVFLLLALFDNASMWMAV
Bor.sp     AMEAADVVVMNDDLRRIAEAVKLSRSTHAVLWQNITLALGIKAVFLVLAVFGNATMWMAV
E.coli     ALETADAALTHNHLRGLVQMIELARATHANIRQNITIALGLKGIFLVTTLLGMTGLWLAV
           *:*:**..: ::. * :.: :.*:: *** : ***.:***:*.:**  :::. : :*:**

R.gel      FADMGASLIVVFNGLRLLRRAKG-
Aci.sp     FADMGASLLVVFNGLRLLRNPERS
Bur.sp     FADMGASLLVVFNGLRLMRTSERS
Bor.sp     FADMGASLLVVVNGLRLLGKAPKA
E.coli     LADTGATVLVTANALRLLRRR---
           :** **:::*. *.***:
```

Fig. S1: CadA/ZntA sequence alignments (Clustal Omega). Conserved key residues and domains are highlighted. The putative transmembrane helices are in bold and underlined. The Histidine rich stretch in *R. gelatinosus* is highlighted in yellow.
Rubrivivax gelatinosus (R. gel), Acidovorax sp. (Aci. sp), Burkholderiales bacterium (Bur. sp) *Bordetella sp. (Bor. sp)* and *Escherichia coli (E. coli)*

B

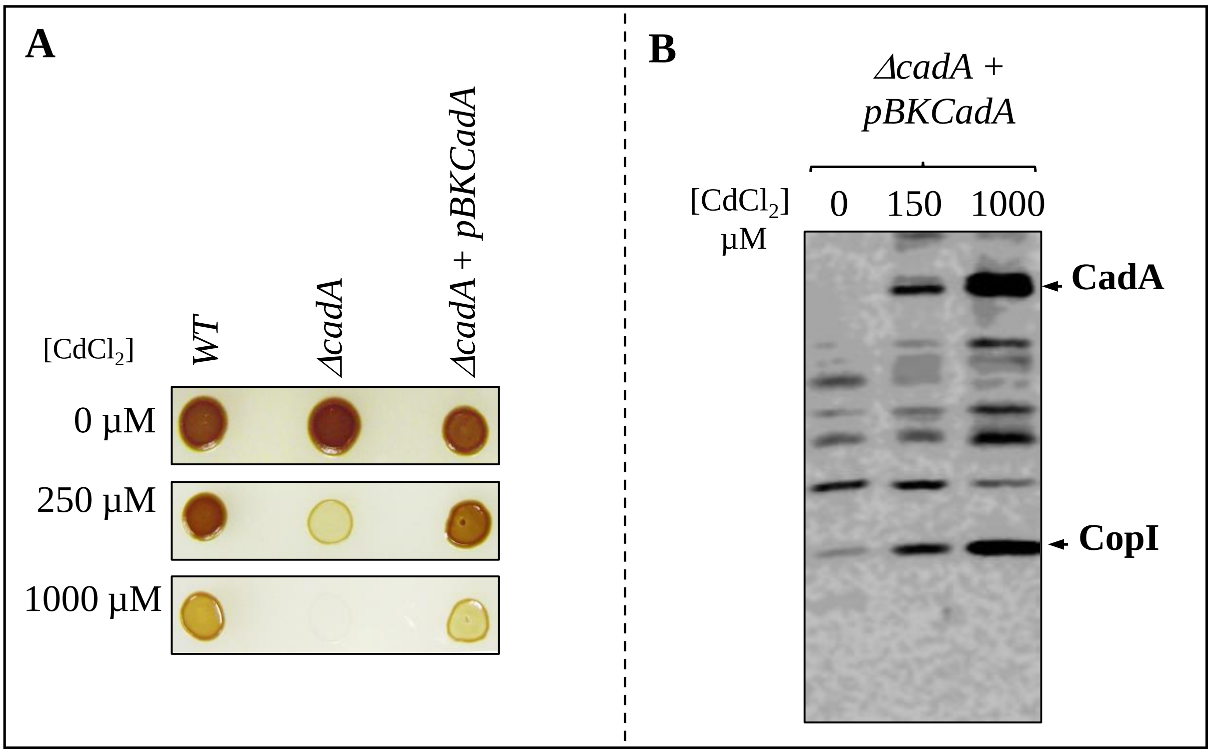

Fig. S2: Complementation of *ΔcadA* strain with the wild-type *cadA* gene (*pBKCadA*).
A- Strains were spoted on solid malate medium supplemented with $CdCl_2$ and incubated under photosynthesis conditions 18 h at 30°C. **B-** Western blot showing the expression and induction of CadA in the complemented strain

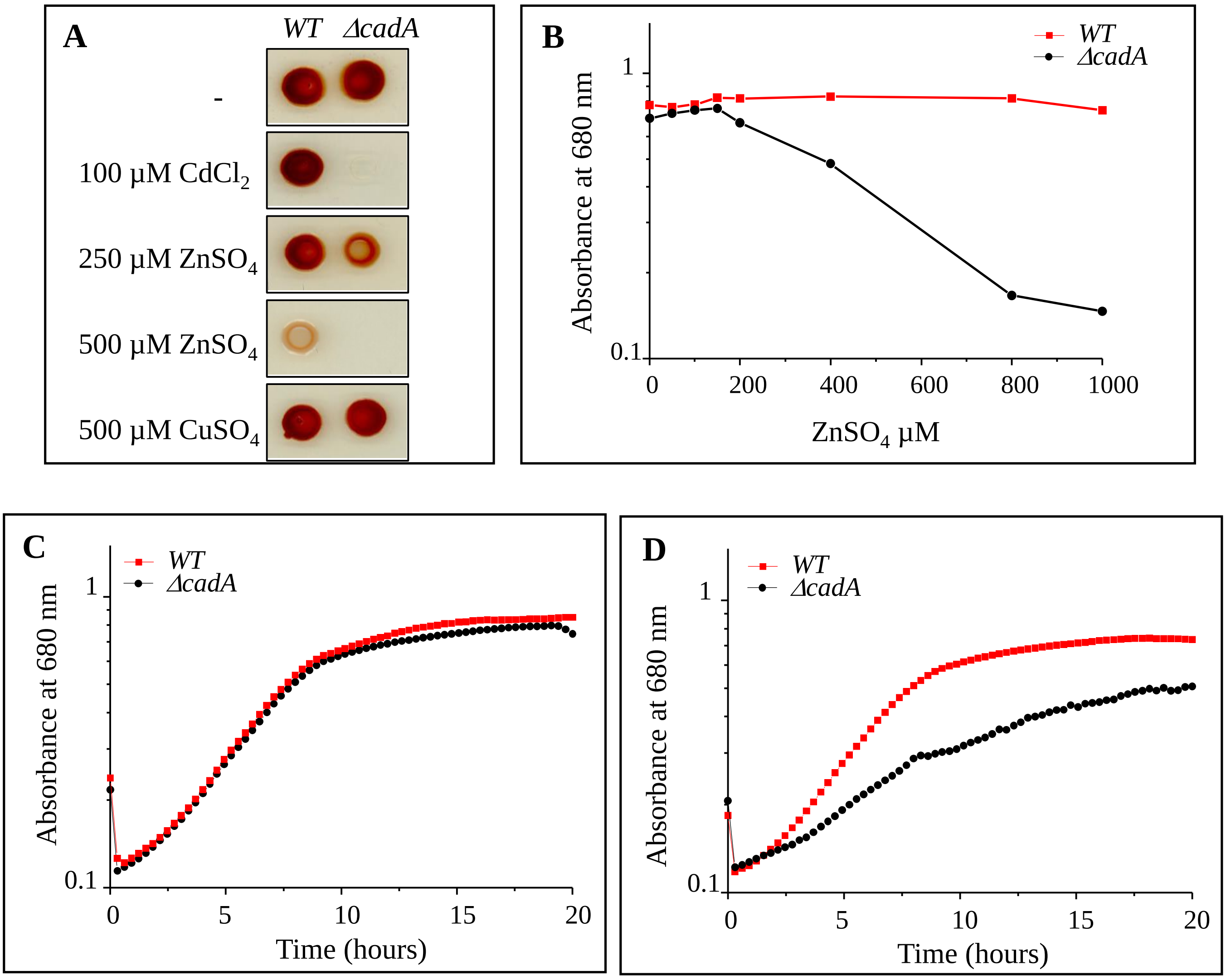

Fig. S3: CadA is also involved in Zn^{2+} tolerance. **A-** WT and *ΔcadA* strain were grown overnight on plates supplemented or not with excess metal as indicated, under respiration conditon. **B-** Growth inhibition of WT and *ΔcadA* strain challenged with increasing $ZnSO_4$. **C-D-** Growth curves of WT and *ΔcadA* strain in malate medium (**C**), or in malate medium supplemented with 400 µM $ZnSO_4$ (**D**).
Cells were grown overnight by aerobic respiration at 30°C.

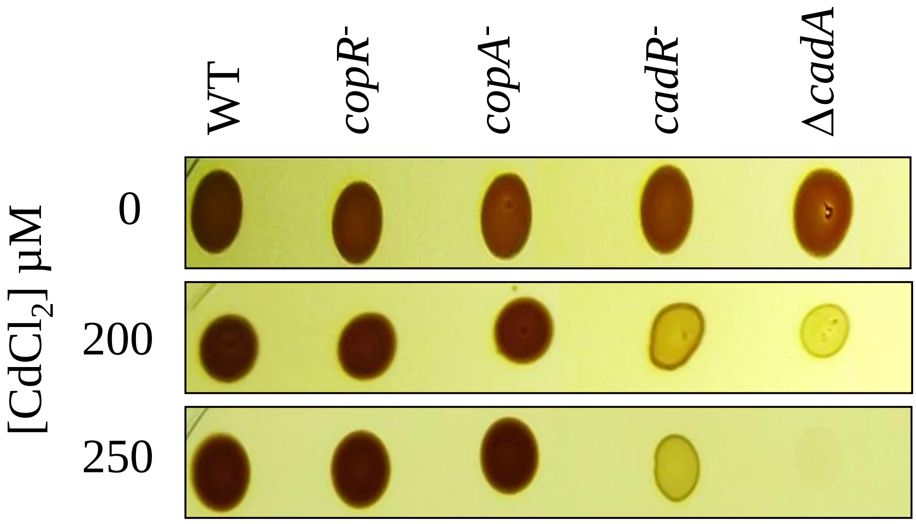

Fig. S4 : Sensitivity of different strains to cadmium. 8 µl of WT, *copR*$^-$ *copA*$^-$ *cadR*$^-$ and Δ*cadA* strains with an OD_{680}=1 were spoted on solid malate medium supplemented with $CdCl_2$ and incubated under photosynthesis conditions for 24 h at 30°C. Δ*cadA* is more sensitive than *cadR*$^-$.

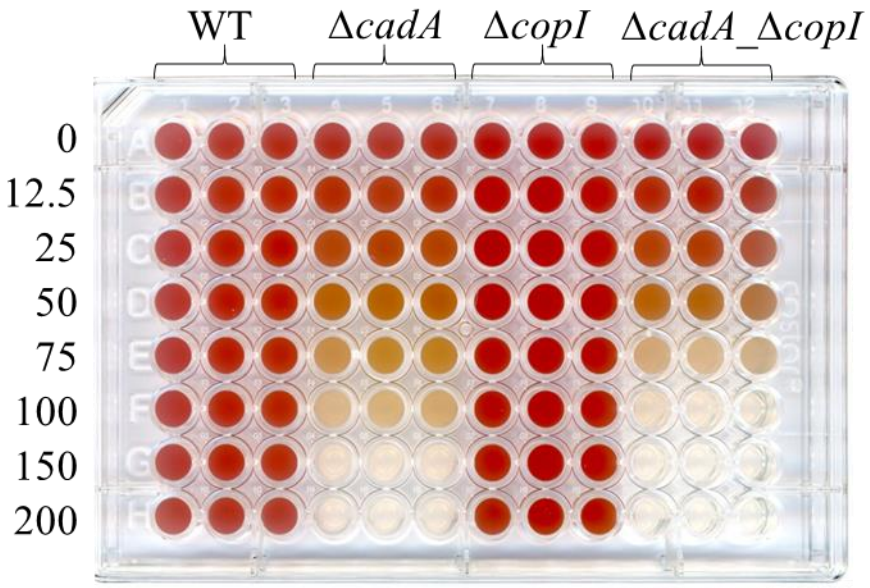

Fig. S5 : Cultures of the wild-type (WT), *ΔcadA*, *ΔcopI* and *ΔcadA-ΔcopI* mutants challenged with increasing $CdCl_2$ concentration after 21 h of growth at 30°C under photosynthesis condition. Three independent experiments have been done for each strain.

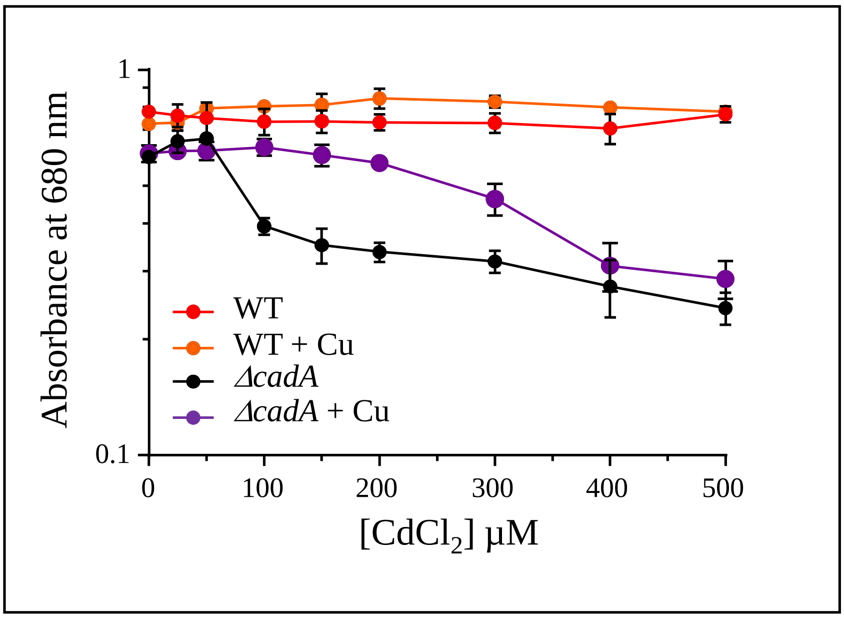

Fig. S6: Effect of $CuSO_4$ on growth in the presence of increasing concentration of $CdCl_2$. Growth inhibition of the wild-type (WT) and *ΔcadA* mutant challenged with increasing $CdCl_2$ concentrations in medium supplemented or not with 200 µM $CuSO_4$. Cells were grown over-night by respiration at 30°C before OD_{680nm} measurement.

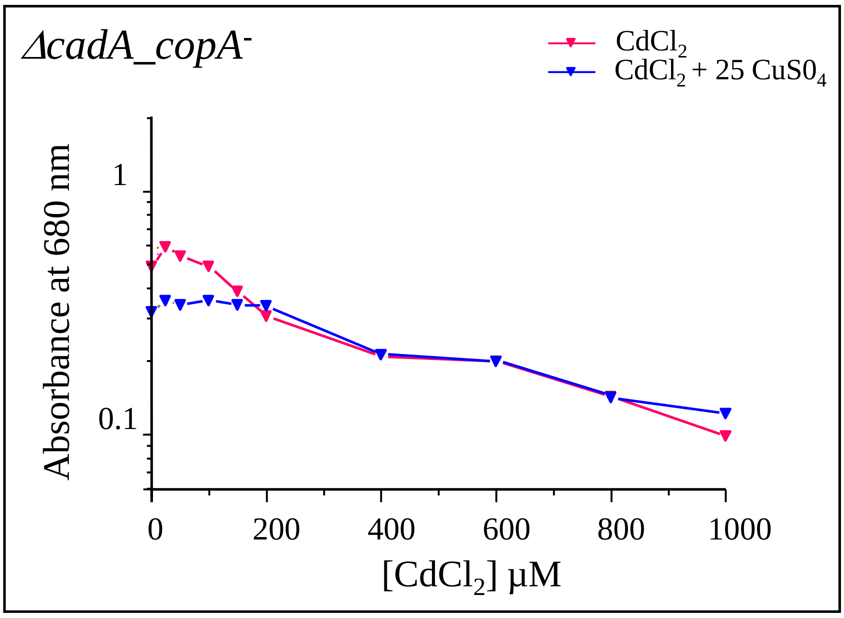

Fig. S7: Effect of $CuSO_4$ on growth in the presence of increasing concentration of $CdCl_2$. Growth inhibition of the *ΔcadA_copA*- double mutant devoid of both CadA and CopA ATPases in Cd^{2+} containing medium supplemented or not with 25 µM CuSO4 . Cells were grown by photosynthesis for 48 h at 30°C

Fig. 2

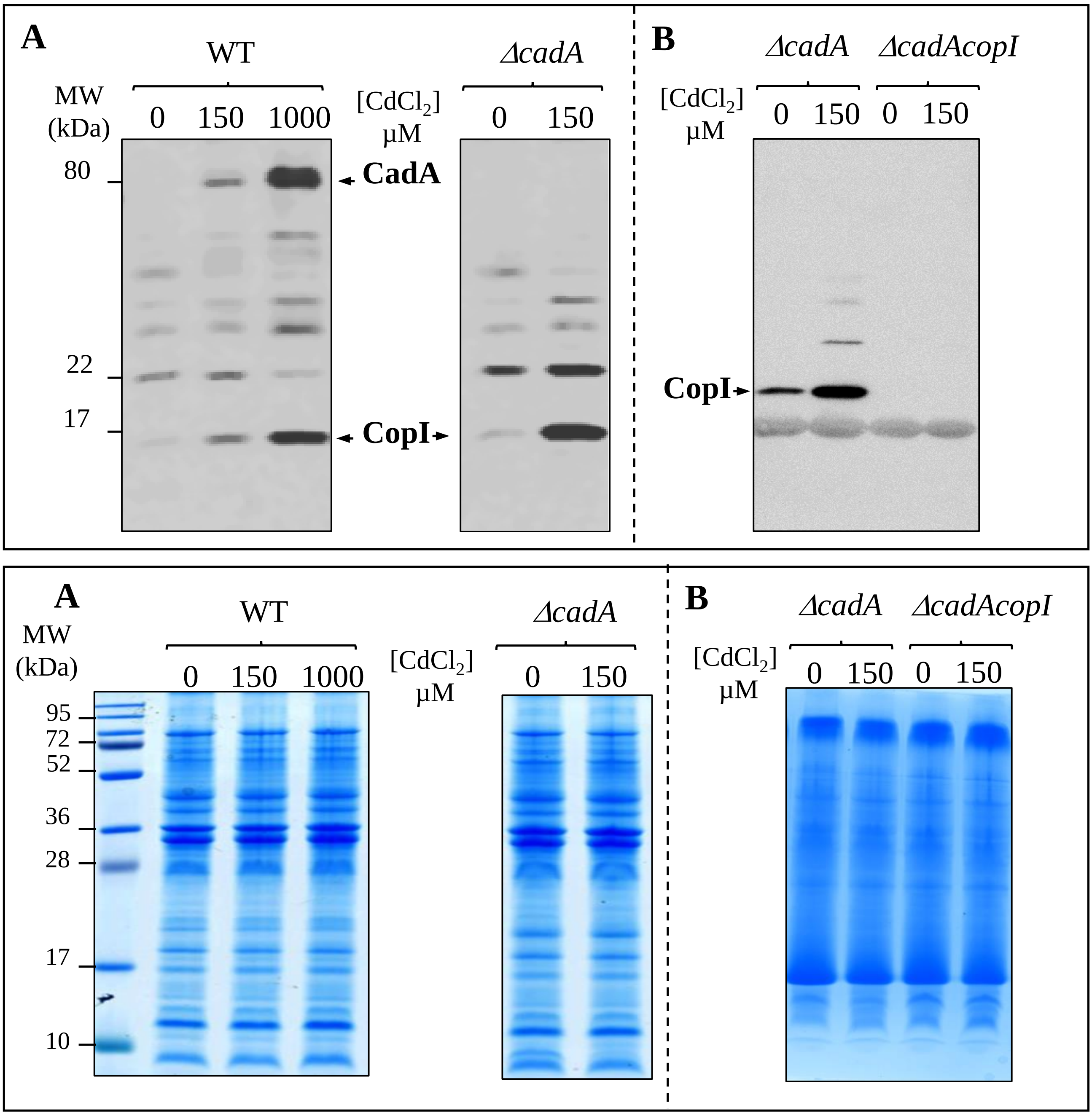
A
WT
ΔcadA
MW (kDa)
0 150 1000
[CdCl2] µM
0 150
80
22
17
CadA
CopI
B
ΔcadA
ΔcadAcopI
[CdCl2] µM
0 150 0 150
CopI
A
WT
ΔcadA
MW (kDa)
0 150 1000
[CdCl2] µM
0 150
95
72
52
36
28
17
10
B
ΔcadA
ΔcadAcopI
[CdCl2] µM
0 150 0 150

Fig. 3

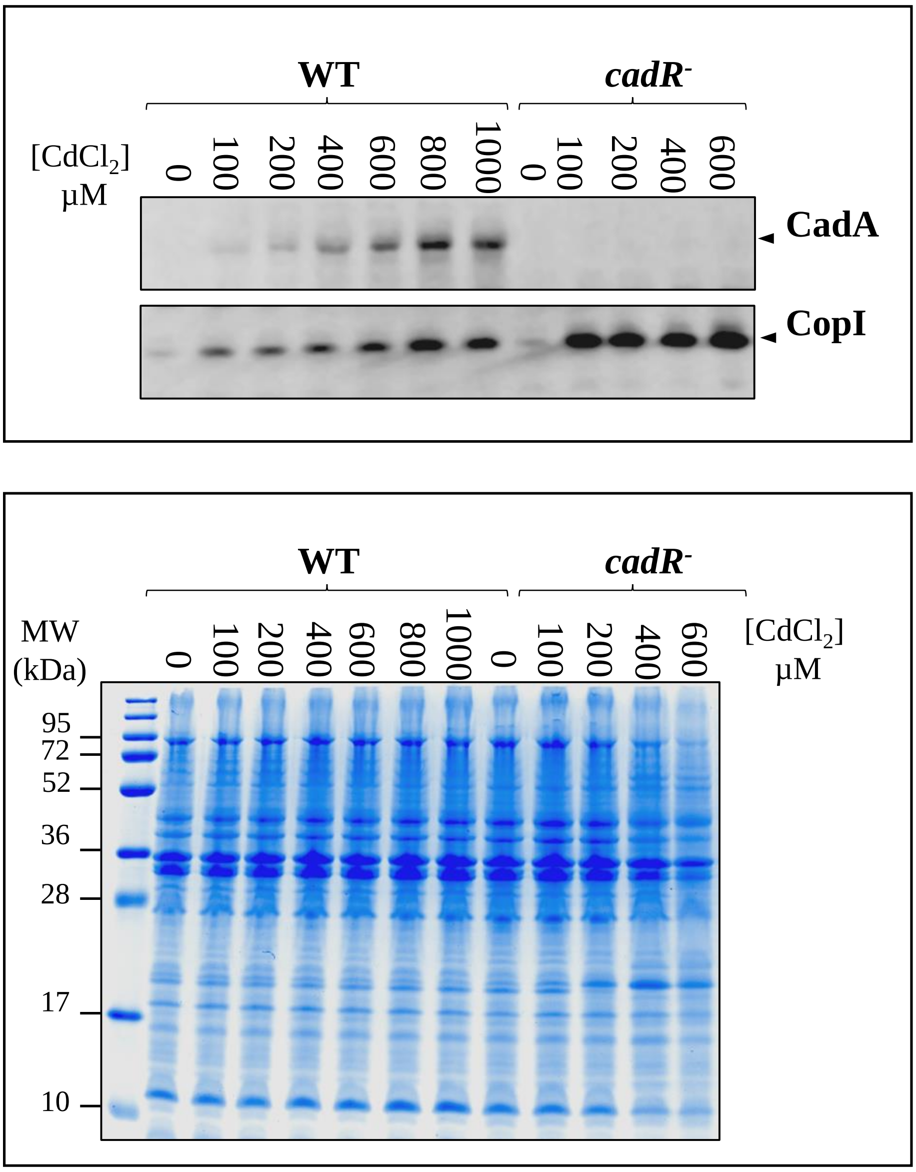
WT
cadR-
[CdCl2] µM
0
100
200
400
600
800
1000
0
100
200
400
600
CadA
CopI
MW (kDa)
95
72
52
36
28
17
10

Fig. 7

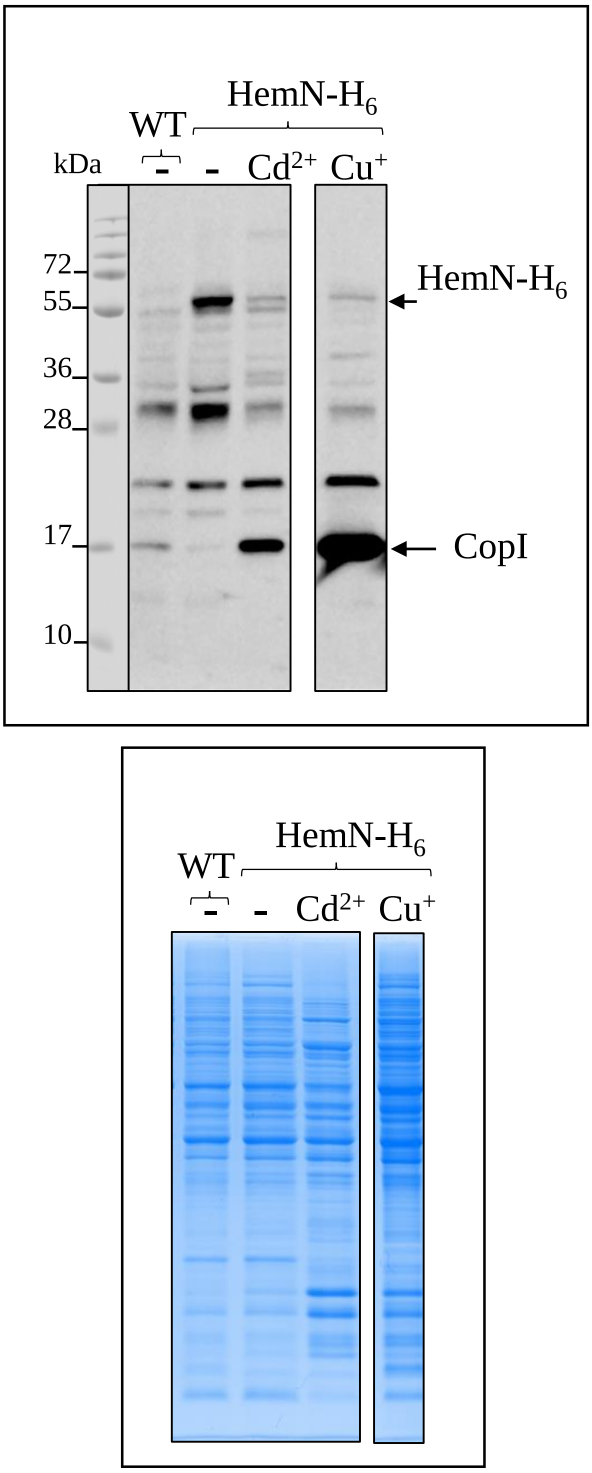
HemN-H6
WT
kDa
-
-
Cd2+
Cu+
72
55
36
28
17
10
HemN-H6
CopI
HemN-H6
WT
-
-
Cd2+
Cu+

Fig. 9

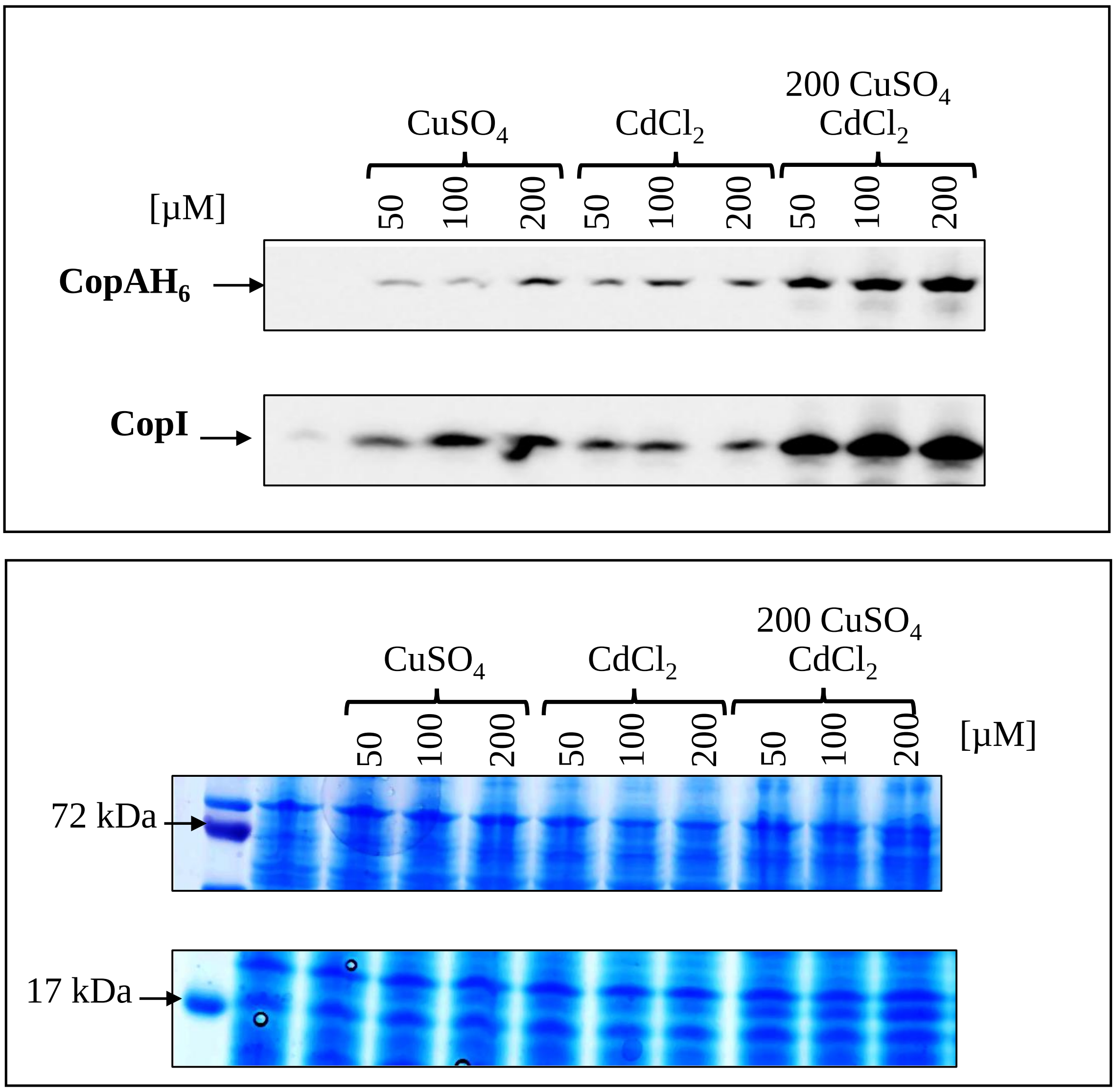

CuSO4
CdCl2
200 CuSO4 CdCl2
[μM]
50
100
200
50
100
200
50
100
200
CopAH6
CopI
CuSO4
CdCl2
200 CuSO4 CdCl2
50
100
200
50
100
200
50
100
200
[μM]
72 kDa
17 kDa
